# A novel peptide derived from *Zingiber cassumunar* rhizomes exhibits anticancer activity against the colon adenocarcinoma cells (Caco-2) *via* the induction of intrinsic apoptosis signaling

**DOI:** 10.1371/journal.pone.0304701

**Published:** 2024-06-13

**Authors:** Kitjasit Promsut, Papassara Sangtanoo, Piroonporn Srimongkol, Tanatorn Saisavoey, Songchan Puthong, Anumart Buakeaw, Onrapak Reamtong, Bodee Nutho, Aphichart Karnchanatat

**Affiliations:** 1 Program in Biotechnology, Faculty of Science, Chulalongkorn University, Pathumwan, Bangkok, Thailand; 2 Center of Excellence in Bioconversion and Bioseparation for Platform Chemical Production, Institute of Biotechnology and Genetic Engineering, Chulalongkorn University, Pathumwan, Bangkok, Thailand; 3 Department of Molecular Tropical Medicine and Genetics, Faculty of Tropical Medicine, Mahidol University, Ratchathewi, Bangkok, Thailand; 4 Department of Pharmacology, Faculty of Science, Mahidol University, Ratchathewi, Bangkok, Thailand; Jadavpur University, INDIA

## Abstract

This paper presents the initial exploration of the free radical scavenging capabilities of peptides derived from protein hydrolysates (PPH) obtained from *Zingiber cassumunar* rhizomes (Phlai). To replicate the conditions of gastrointestinal digestion, a combination of pepsin and pancreatin proteolysis was employed to generate these hydrolysates. Subsequently, the hydrolysate underwent fractionation using molecular weight cut-off membranes at 10, 5, 3, and 0.65 kDa. The fraction with a molecular weight less than 0.65 kDa exhibited the highest levels ABTS, DPPH, FRAP, and NO radical scavenging activity. Following this, RP-HPLC was used to further separate the fraction with a molecular weight less than 0.65 kDa into three sub-fractions. Among these, the F_5_ sub-fraction displayed the most prominent radical-scavenging properties. *De novo* peptide sequencing *via* quadrupole-time-of-flight-electron spin induction-mass spectrometry identified a pair of novel peptides: Asp-Gly-Ile-Phe-Val-Leu-Asn-Tyr (DGIFVLNY or DY-8) and Ile-Pro-Thr-Asp-Glu-Lys (IPTDEK or IK-6). Database analysis confirmed various properties, including biological activity, toxicity, hydrophilicity, solubility, and potential allergy concerns. Furthermore, when tested on the human adenocarcinoma colon (Caco-2) cell line, two synthetic peptides demonstrated cellular antioxidant activity in a concentration-dependent manner. These peptides were also assessed using the FITC Annexin V apoptosis detection kit with PI, confirming the induction of apoptosis. Notably, the DY-8 peptide induced apoptosis, upregulated mRNA levels of caspase-3, -8, and -9, and downregulated Bcl-2, as confirmed by real-time quantitative polymerase chain reaction (RT-qPCR). Western blot analysis indicated increased pro-apoptotic Bax expression and decreased anti-apoptotic Bcl-2 expression in Caco-2 cells exposed to the DY-8 peptide. Molecular docking analysis revealed that the DY-8 peptide exhibited binding affinity with Bcl-2, Bcl-xL, and Mcl-1, suggesting potential utility in combating colon cancer as functional food ingredients.

## Introduction

Cancer poses a significant health challenge for people in all communities, irrespective of their economic status or social standing. The global cancer burden continues to escalate. According to the International Agency for Research on Cancer (IARC), there were an estimated 20 million new cases worldwide and 9.7 million cancer deaths in 2022 [1]. One of the key characteristics of cancer cells is their increased ability to survive compared to normal cells. Reactive oxygen species (ROS) are reported to be tumorigenic due to their ability to enhance cell proliferation, survival, and migration, which can have both beneficial and detrimental effects. ROS serve as secondary messengers that trigger signaling cascades necessary for regular physiological processes such as cell development and differentiation. However, in excess, ROS can damage crucial biomolecules, including lipids, proteins, DNA, and carbohydrates, leading to cellular damage and undesirable pathological outcomes. Studies have established the involvement of ROS in processes such as metastasis, tumorigenesis, and angiogenesis [2].

Antioxidant peptides are garnering increasing attention, particularly in the context of alleviating oxidative stress-related diseases and potentially combating cancer. These peptides, derived from various sources such as plants, animals, and microorganisms, exhibit antioxidant properties that enable them to scavenge reactive oxygen species (ROS) and mitigate oxidative damage in cells [3]. By neutralizing free radicals and stabilizing oxidative processes, antioxidant peptides hold promise in protecting against a range of diseases associated with oxidative stress, including cardiovascular disorders, neurodegenerative conditions, and inflammatory diseases. Moreover, research suggests that antioxidant peptides may offer therapeutic potential in cancer treatment. The ability of these peptides to modulate oxidative stress pathways and promote cellular health has led to investigations into their anticancer properties [4].

The apoptosis pathway is a prospective alternative in cancer therapy since it causes minimal tissue damage, making apoptosis-inducing peptides viable therapeutic possibilities [5]. On the other hand, antioxidant peptides primarily protect cells from oxidative stress. They primarily suppress apoptosis by preserving redox equilibrium, although they can also have an indirect effect on apoptotic pathways. However, their effect on apoptosis varies with cell type and environmental factors. Some studies have revealed an antioxidant peptide that inhibits apoptosis. Rapeseed protein prevented H_2_O_2_-induced apoptosis in human umbilical vein endothelial cells (HUVECs); this peptide could specifically reduce the expression of Bax and caspase-3 while increasing the expression of Bcl-2 [6]. Another example is a new antioxidant peptide discovered in the epidermis of *Odorrana margaretae* (OM-GL15). OM-GL15 was shown to protect epidermal cells against UVB-induced apoptosis by increasing Bcl-2 and decreasing caspase-3, caspase-9, and Bax. It was proposed that OM-GL15 may be developed as a photodamage inhibitor for the cosmetics sector [7]. Further research is needed to understand the complex interactions between antioxidant peptides and anticancer through the apoptosis pathway, offering potential for novel therapeutic strategies against oxidative stress-related diseases.

Recently, various plant has been shown to be good sources of proteins for obtaining antioxidant peptides due to their high bioactivities. Bioactive peptides are usually found as oligopeptides inside the principal sequences of the original proteins. They stay inactive until they are released through processes such gastrointestinal digestion, fermentation, or enzymatic hydrolysis. These released peptides, which have a small molecular weight, have excellent digestion and improved bioavailability compared to intact proteins. As a result, they play a role in different physiological functions [8]. Despite significant advancements, a research gap persists in exploring plant-derived bioactive peptides, particularly in identifying novel sources with unique therapeutic potential. This underscores the necessity for studies aimed at uncovering peptides from underexplored botanical species. Phlai, the common name for *Zingiber cassumunar* Roxb., is an herbaceous member of this family known for its rhizomes, the component growing underground. Widely used in Thailand to address various ailments including arthritis, sprains, rheumatism, inflammation, respiratory problems, and musculoskeletal, menstrual, or gastrointestinal pain [9, 10]. Phlai contains compounds such as quinones, curcuminoids, benzaldehydes, phenylbutenoids, sesquiterpenoids, and essential oils containing monoterpenoids. Both these components and extracts from Phlai have demonstrated anti-inflammatory, antioxidant, anti-cancer, neuroprotective/neurotrophic, and antibacterial/antifungal properties [11, 12]. However, it is noteworthy that data regarding anticancer peptides derived from Phlai has not yet been reported.

The investigation aims to assess the antioxidant capabilities of Phlai peptides and explore their potential applications in anticancer therapy, particularly through the investigation of apoptosis mechanisms. Through our research, we have gained valuable insights into the ability of Phlai peptides to mitigate oxidative stress and improve overall antioxidant efficacy. In order to assess these properties, we performed widely employed antioxidant assays including 2, 2’-azinobis (3-ethylbenzothiazoline-6-sulfonic acid) (ABTS), 2, 2’-diphenyl-1-picrylhydrazyl (DPPH), nitric oxide (NO), and ferric ion-reducing antioxidant power (FRAP). These assays serve as robust methods to assess the antioxidant capabilities of Phlai peptides, contributing to our understanding of their potential applications in combating oxidative stress. [13]. Additionally, we examined gene and protein expression related to the apoptosis pathway, alongside utilizing computational methods to predict 3D structures and conduct molecular docking studies. These analyses elucidated the peptides’ potential for combating colon cancer by evaluating their binding affinity towards target proteins in the apoptosis pathway, highlighting their promising applications in the pharmaceutical sector.

## Materials and methods

### Biological material

Fresh *Z*. *cassumunar* rhizomes were obtained from Chatuchak Market in Bangkok, Thailand. The samples were placed in storage in darkness at a temperature of 4°C at the laboratory until required for experimentation. The American Type Culture Collection (ATCC, Manassas, VA, USA) supplied the human intestinal epithelial cell line, Caco-2 cells (ATCC^®^ HTB-37^™^) which were then cultured in RPMI 1640 (Roswell Park Memorial Institute: RPMI) with 10% fetal bovine serum (FBS) provided by Gibco (Rockville, MD, USA).

### Chemicals

Acrylamide, ammonium persulfate (APS), ammonium sulfate ((NH_4_)_2_SO_4_), L-ascorbic acid, 2,2’-Azobis(2-methylpropionamidine) dihydrochloride (ABAP), ABTS, bovine serum albumin (BSA), Coomassie Brilliant Blue G, curcumin, 3-(4,5-dimethyl-2-thiazolyl)-2,5-diphenyl-2H-tetrazolium bromide (MTT), 2’-7’ dichlorodihydrofluorescein diacetate (DCFH-DA), DPPH, Dimethyl sulfoxide, (DMSO), ferric trichloride hexahydrate (FeCl_3_.6H_2_O), fetal calf serum (FCS), disodium hydrogen phosphate (Na_2_HPO_4_), monosodium dihydrogen orthophosphate (NaH_2_PO_4_), hydrochloric acid (HCl), *N*,*N*’-methylenebis(acrylamide), naphthylethylenediamine dichloride (NED; C_12_H_16_Cl_2_N_2_), pancreatin from porcine pancreas (8 × USP specifications), pepsin from porcine gastric mucosa (≥250 U/mg), potassium persulphate (K_2_O_8_S2), quercetin, Sodium acetate, (CH_3_COONa), sodium chloride (NaCl), sodium dodecyl sulfate (SDS), sodium hydroxide (NaOH), sodium nitroprusside (SNP; C_5_FeN_6_Na_2_O), sulphanilamide (C_6_H_8_N_2_O_2_S), 2,4,6-tri(2-pyridyl)-S-triazine (TPTZ), and tris hydrochloride (Tris-HCl) were obtained from Sigma-Aldrich, Merck (St. Louis, MO, USA). Thermo Fisher Scientific (San Jose, CA, USA) supplied the ethanol, acetonitrile (ACN), formic acid, *N*,*N*,*N*’,*N*’-tetramethyl ethylenediamine (TEMED), trifluoroacetic acid (TFA), and Tween 20 all of which were of chromatographic grade. All chemicals used in the course of this research were of analytical grade.

### Crude protein extraction from Phlai rhizomes

A modified version of Inthuwanarud *et al*.’s method was utilized to extract protein from Phlai rhizomes. The rhizomes (1.5 kg wet weight) underwent peeling, slicing, and homogenization in 5 L of PBS (0.15 M NaCl with 20 mM phosphate buffer, pH 7.2) using a blender (HR 2061; Philips, Bahasa, Indonesia) [14]. The mixture was agitated overnight at 4°C using an agitator (Model RW-20; IKA. Labortechnik, Staufen, Germany). The resulting suspension was filtered through double-layered cheesecloth and centrifuged for 30 minutes at 15,000 × *g*. Ammonium sulfate was added to the supernatant, stirred to 80% saturation, and left overnight at 4°C with continued stirring. After centrifugation at 15,000 × *g* for 30 minutes to collect the precipitate, the supernatant was discarded. The obtained pellet was dissolved in 20 mL of PBS, dialyzed using tubing with a 3,500 Dalton cut-off (SnakeSkin, Thermo Scientific Co., Ltd., USA), and finally freeze-dried as the crude protein preparation.

### Phlai protein hydrolysis using pepsin and pancreatin

Initially, Phlai proteins were used as the substrate to produce the protein hydrolysate, following the method described by Petsantad *et al*. [15] Briefly, freeze-dried protein is dissolved in DDI water at a ratio of 10% (w/v) for 30 min to solubilize and evenly disperse the protein. Sequential enzyme digestion was carried out with pepsin/substrate 1:20 (w/w) enzyme-substrate (E/S) ratios, with the pH adjusted to 2.5 using 1 M HCl. The hydrolysis occurred at 37°C for three hours with constant shaking at 180 rpm. Inactivation was achieved by raising the pH to 7.5 with 1 M NaOH. Subsequently, pancreatin was added by pancreatin/substrate 1:20 (w/w) E/S ratio and incubated for three hours at 37°C with shaking at 180 rpm. The enzyme reaction was terminated by heating to 80°C for 20 minutes. The hydrolysates were then clarified through centrifugation at 15,900 × *g* for 30 minutes at 4°C. The antioxidant activity of the supernatant was then measured. The protein concentrations for each of the protein hydrolysates could be determined using the Bradford procedure under the BSA standard. The absorbance was then measured at 595 nm using a spectrophotometer (Multiskan GO; Thermo Fisher Scientific, Waltham, MA, USA) [16].

### Antioxidant capacity measurement

#### ABTS radical scavenging activity assay

The ABTS•+ radical scavenging assay was conducted following a modified procedure proposed by Aursuwanna *et al*. [17] A solution of 7 mM ABTS reacted with potassium persulphate at a molar ratio of 1:0.5, resulting in a final concentration of 2.45 mM, and incubated for 12 hours at room temperature to form ABTS•+ radical cations. The solution was diluted with phosphate buffer solution (0.1 M, pH 7.4) to attain an absorbance of 0.70 (± 0.02) at 734 nm (A_734_) and equilibrated at 30°C. Test samples were mixed with the ABTS•+ solution at a ratio of 1:30 (v/v), followed by a 10-minute incubation in darkness. Absorbance was then measured at A_734_ using a microplate reader, with measurements taken in triplicate and ascorbic acid used as the positive control.

#### DPPH radical scavenging activity assay

The approach of Saisavoey *et al*. [18] with slight modification, was employed to conduct the DPPH^•^ free radical scavenging assay, whereby a DPPH^•^ solution of 100 μM in methanol was introduced to each sample at a ratio of 1:4 (v/v), or 80 μL of sample to 320 μL of DPPH radical solution. The resulting mixture underwent 15 minutes of incubation at room temperature in darkness, before being placed in a centrifuge for 5 minutes at 15,000 × *g*. A microplate reader was then used to take absorbance measurements at 517 nm (A_517_). Ascorbic acid served as the positive control.

#### Nitric oxide radical scavenging assay

The approach of Suttisuwan *et al*. [19] was employed to assess the nitric oxide (NO) radical scavenging ability. Initially, nitric oxide radicals were produced by using a sodium nitroprusside solution to create nitrite ions, which could be quantified *via* the Griess reaction. Different dilutions of the protein hydrolysates in the amounts of 25 μL were added to 10 mM SNP in phosphate buffer (pH 7.2), whereupon the mixture underwent incubation for 150 minutes at room temperature. This was followed by the addition of 100 μL of 0.33% sulphanilamide in 20% acetic acid and this mixture was permitted to stand for 5 minutes to allow diazotization to be completed. Finally, 0.1% NED was added before further incubation for 30 minutes at room temperature. Absorbance measurements were taken at 546 nm (A_546_), while the positive control used was curcumin.

#### FRAP (Ferric Reducing Antioxidant Power) assay

In the FRAP assay, Fe^3+^- TPTZ is reduced to a blue colored Fe^2+^-TPTZ, following a method based on that of Benzie and Strain [20]. Initially, TPTZ prepared at a concentration of 10 mM in 40 mM HCl, was added to 20 mM FeCl_3_.6H_2_O and 0.3 M sodium acetate buffer (Ph 3.6) in the ratio of 10:1:1 (v/v/v) in order to produce the FRAP reagent. The solution obtain is the color of straw, and is placed in a water bath for incubation at a temperature of 37°C. This fresh FRAP reagent (3.0 ml) is then introduced to the samples (100 μl), shaken, and incubated for 4 minutes. The absorbance is measured for this reaction mixture at 593 nm (A_593_) with a spectrophotometer, and compared to the FRAP reagent blank. The capacity of the samples for ferric ion reduction indicates the antioxidant capacity, expressed in terms of ascorbic acid equivalent (mg/g).

#### Calculations of percentage inhibition

The radical scavenging percentage was determined *via* Eq (1) as shown below:

$$\frac{\left[\left(\text{Abs}\;\text{control}–\text{Abs}\;\text{blank}\right)–\left(\text{Abs}\;\text{sample}–\text{Abs}\;\text{background}\right)\right]}{\left(\text{Abs}\;\text{control}–\text{Abs}\;\text{blank}\right)}\times 100,$$ (1)

where Abs control is the control absorbance (no sample), Abs blank is the deionized water absorbance, Abs sample is the Phlai protein hydrolysis (PPH) absorbance, and Abs background is the samples’ color absorbance. The IC_50_ value, or the PPH concentration required to achieve 50% antioxidant activity inhibition, can be evaluated with GraphPad Prism v. 6.01 for Windows (GraphPad Software Inc., San Diego, CA, USA).

### Isolation and enrichment of bioactive peptides

#### Ultrafiltration

The PPH fraction achieving the highest level of free radical scavenging having been separated using the molecular weight cut-off membranes (Pellicon XL Filter; Merck Millipore, Billerica, MA, USA) was selected for additional ultrafiltration in order to create five new sub-fractions of different sizes: MW ≥ 10 kDa, MW 5–10 kDa, MW 3–5 kDa, MW 0.65–3 kDa, and MW < 0.65 kDa.

#### RP-HPLC (Reverse Phase High Performance Liquid Chromatography)

In this stage, the antioxidant protein fractions which offered the greatest activity underwent further filtration using a 0.45 μm nylon membrane (Whatman, GE, Buckinghamshire, UK) prior to separation using RP-HPLC (Spectra System, Thermo Fisher Scientific, San Jose, CA, USA) with a Luna 5U 100A column (4.6 mm × 250 mm, Luna 5 μM, Phenomenex, Torrance, CA, USA) generating a linear gradient in three phases of 100: 0% (v/v) A: B declining to 90: 10 (v/v) A: B at 18 minutes, and subsequently to 65: 35 (v/v) A: B at 30 minutes, and eventually achieving 55: 45 (v/v) A: B at 40 minutes when the constant flow rate was 0.7 mL/min. The composition of mobile phase A was assessed as 0.1% (v/v) TFA, while phase B was 70% (v/v) ACN in 0.05% (v/v) TFA. Chromatographic analysis was also carried out using ChromQuest software (Thermo Fisher Scientific, San Jose, CA, USA). To do this, the temperature was 25 °C, and an injection volume of 20 μL was used with injected samples offering a protein concentration in the range of 1.50 to 2.00 mg protein/mL. The peptide peaks were eluted and assessed at 280 nm, thus enabling determination of the amino acid sequences for each purified peptide *via* electrospray quadrupole time-of-flight tandem mass spectrometry: (ESI)-Q-TOF-MS/MS.

### Identification and synthesis of peptides

The identification of the peptide contained within the RP-HPLC fraction (F_5_ sub-fraction), exhibiting the highest antioxidant activity, was achieved through the utilization of an electrospray ionization and an UltiMate 3000 nano-liquid chromatography system (Thermo Fisher Scientific), coupled with a micrOTOF-Q electrospray ionization quadrupole time-of-flight MS (Bruker Daltonics, Billerica, MA, USA). Subsequently, the data were analyzed using a de novo sequencing approach. For sample injection, mobile phase A comprised 2% acetonitrile and 0.1% formic acid in water, while mobile phase B consisted of 0.1% formic acid in acetonitrile, flowing at a rate of 300 nL/min for 60 minutes. Data acquisition was managed using Hystar software (Bruker Daltonics Data). The peptide spectra were obtained within two mass ranges: m/z 400–3,000 and 50–1,500. LC-MS/MS data were utilized for *de novo* sequencing, and the MS data files were scrutinized using Mascot database software (Matrix Science, London, UK) against the NCBI database. The resulting amino acid sequence was then evaluated *via* the NCBI database using the BLASTp program, allowing the identification and statistical assessment of matches found.

Following peptide identification, further synthesis was carried out *via* Fmoc solid-phase synthesis utilizing an Applied Biosystems Model 433A Synergy peptide synthesizer (Applied Biosystems, Foster City, CA, USA). The peptide purity was subsequently assessed using analytical mass spectrometry employing a quadrupole ion trap Thermo Finnigan^™^ LXQ^™^ LC-ESI-MS (San Jose, CA, USA) connected to a Surveyor HPLC (Thermo Fisher Scientific, San Jose, CA, USA). The HPLC results indicated that the synthetic peptides should exhibit a purity of no less than 98%. The sequences were determined to be Asp-Gly-Ile-Phe-Val-Leu-Asn-Tyr (DGIFVLNY or DY-8; m/z = 940.478) and Ile-Pro-Thr-Asp-Glu-Lys (IPTDEK or IK-6; m/z = 702.367), with a molecular weight of 1,014.20 Da and HPLC purity exceeding 98%. The antioxidant capabilities of these synthetic peptides were also evaluated.

### Bioinformatics tools supporting the annotation of peptide properties

To guide future potential applications, it is necessary to understand the properties of different antioxidant peptides. Data are available online, so in this case, the potential profiles of DY-8, and IK-6 can be found using the BIOPEP database, which is available at: www.uwm.edu.pl/biochemia/index.php/en/biopep. BIOPEP offers the additional advantage of classifying the possible sensory qualities of different peptides, which is useful for the food industry. The peptide solubility can be assessed using the Innovagen server (www.innovagen.com/proteomics-tools), while hydrophobicity information is available from Peptide2 (www.peptide2.com). Meanwhile, *in silico* toxicity of the peptides can be calculated using the ToxinPred server (crdd.osdd.net/raghava/ toxinpred), which uses an SVM threshold score below zero to indicate non-toxicity, or AllerTOP (http://www.ddg-pharmfac.net/AllerTOP/), which makes use of auto cross covariance (ACC) transformation to make *in silico* predictions concerning toxicity and allergenic properties. The 3D structure of peptides can be assessed *in silico* using the PEP-FOLD tool V3.5 (http://bioserv.rpbs.univ-paris-diderot.fr/services/PEP-FOLD3/) which takes into consideration the amino acid sequences in making the prediction, while the 3D structure images were created with Discovery Studio software 2016 (Accelrys Software Inc., San Diego, CA, USA).

### Cellular antioxidant activity assay (CAA)

#### DY-8 and IK-6 cytotoxicity

Cytotoxic activity was determined *in vitro* for the DY-8 and IK-6 peptides, by evaluating the free scavenging activity in the context of the Caco-2 (human adenocarcinoma colon) cell line. Cell suspensions contained within complete medium [CM; RPMI with 10% (v/v) FCS] underwent dilution before plating in 200 μL/wells in 96-well plates, achieving a final absorbance of 1.0 at 540 nm (A_540_). This was followed by incubation in 5% carbon dioxide for 72 hours at a temperature of 37°C. Fresh CM was then used to replace the cell culture medium, introducing differing concentrations of the DY-8 and IK-6 peptides, whereupon further incubation of the resulting mixture took place for 72 hours. Each well then received 10 μL of 5 mg/mL MTT in normal saline solution prior to further incubation for 4 hours before removal of the media. The insoluble purple formazan crystals were then dissolved using 150 μL of DMSO per well, and a microplate reader spectrophotometer was used for the absorbance measurement at 540 nm. This result was determined to be in direct proportion to the number of viable cells, thus allowing the calculation of the relative percentage cell viability using Eq (2) as shown:

$$\text{Cell}\;\text{survival}\;\text{rate}\left(\%\right)=Abs_{sample}/Abs_{control}\times 100$$ (2)

in which a rate of 100% cell survival was set as the control, and calculation of the IC_50_ value was based on the obtained data through the use of the GraphPad Prism software version 6.01. Each assay was carried out in triplicate.

#### CAA

The in vitro cellular antioxidant activity of the DY-8 and IK-6 peptides was assessed using the method outlined by Wolfe and Liu [21]. Caco-2 cells were seeded at a density of 6 × 10^4^ cells per well in a 96-well plate and incubated for 24 hours until reaching 90–100% confluence. Outer wells were left unused to prevent bias. After removing the growth medium, cells were washed with sterile 20 mM PBS (pH 7.4) to remove dead or non-adherent cells. Each well was then treated with varying concentrations of DY-8 or IK-6 peptides (100 μL), along with 50 μL of 50 μM DCFH-DA probe solution, followed by one hour of incubation at 37°C. After removing the treatment solutions and rinsing the cells with PBS, each well received 100 μL of 500 μM ABAP solution, except for blank and negative control wells. Fluorescence was measured at 528 nm excitation and 485 nm emission wavelengths every 5 minutes for 90 minutes using a microplate reader at 37°C. The percentage decrease, referred to as the CAA unit, was calculated using Eq (3).

$$\text{CAA}\;\text{Unit}=\%\text{reduction}=\left(1-AUC_{sample}/AUC_{control}\right)\times 100$$ (3)

in which *AUC*_*sample*_ represents the area under the curve showing sample fluorescence against time, and *AUC*_*control*_ represents the area integrated from the quercetin control curve obtained from four different experiments with results obtained in triplicate and shown in the form of mean ± standard error.

### Quantitative real-time reverse transcription-polymerase chain reaction (qPCR)

The qPCR analysis required Caco-2 cells to be cultured with different concentrations of DY-8 and IK-6 peptides using 6-well plates for durations of 24 hours and 48 hours. The cells were then harvested through EDTA-free trypsinization and rinsed thoroughly two times in cold PBS. Subsequently, a MasterPure RNA Purification Kit (Epicentre, USA) was used to obtain the RNA from the cells in line with the instructions of the manufacturer. The reverse transcription of the total RNA (1 μg) was conducted with Oligo-dT primers using a precision nanoScript II reverse transcription kit (PrimerDesign, Camberley, UK). A NanoDrop 2000 UV-Vis spectrophotometer (Thermo Fisher Scientific, Inc.) was then used to measure the RNA concentration at 260 nm. The different primers used in the qPCR assays are shown in S1 Table, while all assays were conducted using MyGo Pro^®^ Real time PCR apparatus (IT-IS International Ltd., Stokesley, UK). Meanwhile, the 20 μL PCR reaction was carried out using 1 μL of cDNA, 1 μL of each primer (10 mM), 7 μL of ultrapure water, and 10 μL of 2× qPCRBIO SyGreen Mix (PCR Biosystems Ltd., London, UK). The reaction was started through a 2-minute activation step at 95°C, whereupon 40 ten-second cycles followed at 95°C, and then cycles of 20 seconds at 60°C, and 30 seconds at 72°C, to form a melting curve in the range of 55–95°C using one minute for each stage. Determination of the The cycle threshold (C_t_) was determined through experimentation in triplicate, whereupon the data were normalized using β-actin as the internal control. The obtained C_t_ value could then be employed to determine the relative gene expression *via* the method of Livak and Schmittgen [22], as shown below in Eq (4):

$$\text{Relative}\;\text{gene}\;\text{expression}=2^{-\Delta \Delta Ct}$$ (4)

where ΔΔC_t_ represents the heightened target gene threshold cycle when the threshold cycle is rising for the housekeeping gene (β-actin). Application of the formula generates a value, which is indicative of no change if the value equals one. If the value is less than one, gene expression is understood to have been reduced, whereas a value exceeding one indicates an increase.

### Apoptosis

To examine apoptosis, dual staining of cells with Annexin V-FITC and propidium iodide (PI) was conducted following the protocol of the Annexin V-FITC/PI detection kit (BioLegend Inc., San Diego, CA, USA). Caco-2 cells were initially seeded in 25 cm^2^ culture flasks (1 × 10^7^ cells/flask) in CM with 2.0 mM L-glutamine. After overnight incubation, DY-8 and IK-6 peptides at various concentrations and 0.5 mg/mL of doxorubicin as a positive control were added. Incubation continued for 24, 48, or 72 hours at 37°C. Cells were harvested using a scraper, washed in cold phosphate-buffered saline (pH 7.2) with 1% (v/v) FCS, and centrifuged. The cell pellets were then resuspended in Annexin V-binding buffer (100 μL), and 100 μL of the cell suspension was mixed with 5 μL of PI solution and 2.5 μL of Annexin V-FITC in a microcentrifuge tube. After vortexing and incubation in darkness for 15 minutes at room temperature, 200 μL of Annexin V-binding buffer was added. Apoptosis was detected using a Cytomics EC500 MPL flow cytometer (BD FACSCalibur, BD Biosciences, Singapore), and data were analyzed using FlowJo software version 7 (FlowJo LLC, Ashland, OR) [23].

### Western blot analysis

The expression levels for the apoptosis signaling pathway protein caspase-3, cleaved caspase-3, caspase-9, Bax, and Bcl-2 were analyzed *via* Western blot analysis. To do so involved treating the Caco-2 using different concentrations of the DY-8 and IK-6 peptides over a duration of 48, and 72 hours. Harvested cells were subsequently lysed using NP40 Cell Lysis Buffer supplemented with a protease inhibitor cocktail. Protein concentrations were measured using the BSA method. In every treatment scenario, the protein extract was exposed in equal amounts to electrophoresis with 12% sodium dodecyl sulfate poly-acrylamide gel electrophoresis (SDS-PAGE) gel before moving to polyvinylidene fluoride (PVDF) membranes (Millipore, Bedford, MA, USA). The membranes were then blocked with 3% skimmed milk (w/v) in tris-buffered saline TBST (1× TBS buffer and 0.5% Tween 20) for one hour while maintaining room temperature. The primary antibody (S1 Text) was used to probe the membranes, followed by incubation with gentle overnight shaking at 4°C. The secondary antibodies (1:5000) were then washed thrice in TBST, and added to the membranes for 1 hour of room temperature incubation. The three washes in TBST were then repeated before the bands were developed using enhanced chemiluminescence HRP substrates (GeneDirex, Taiwan). Band images were then collected using an ImageQuant LAS 500 (GE Healthcare, Uppsala, Sweden), while the variations in protein expression were evaluated *via* ImageJ software.

### Preparation of the system and molecular docking

Analysis of molecular docking establishes the binding modes for the DY-8 peptide to the three principal Bcl-2 families of antiapoptotic proteins: Bcl-2, Bcl-xL, and Mcl-1. The Protein Data Bank provided the details of the 3D structures of Bcl-2, Bcl-xL, and Mcl-1 based on the respective Protein Data Bank accession codes of 2XA0 [24], 3FDL [25], and 2NLA [26]. The tleap module used in AMBER20 [27] was employed to create the peptide structure, whereupon the 3D structure of the DY-8 peptide underwent energy-minimization via 2000 steps of steepest descent and 1000 steps of conjugated gradient methods, on the basis of the implicit solvent calculation (igb = 2). Docking simulation was then carried out *via* the HDOCK webserver (http://hdock.phys.hust.edu.cn/) [28] to examine the molecular docking interactions of the prepared peptides and proteins. Those docked complexes which achieved the lowest HDOCK scores were then selected for further analysis of the protein-peptide interactions *via* the Discovery Studio Visualizer (BIOVIA, San Diego, CA, USA) and also the ChimeraX program [29] which enables the creation of 3D visualizations.

### Statistical analysis

Assays were all performed in triplicate, while data were shown as mean ± standard deviation (S.D.). Analysis relied upon the Statistical Package for Social Sciences (SPSS) version 15.0 (SPSS, Inc., USA). Data from the experiments were evaluated *via* Duncan’s multiple range tests in order to compare data at a statistical significance level of p < 0.05, or in some cases, p < 0.01.

## Results and discussion

### Pepsin-pancreatin hydrolysis of Phlai protein isolates

For rapid screening of new foods or delivery systems, in vitro digestion techniques can replace animal or human digestion models, offering cost-effective, fast, and safe testing procedures. Unlike industrially produced bioactive peptides, which tend to degrade rapidly and lose their properties upon digestion, the preparation of protein hydrolysates is often employed in vitro gastrointestinal (GI) digestion models. This process is known to generate antioxidant peptides through enzymatic digestion in the gastrointestinal tract [30]. Many oligopeptides with 2–10 amino acids can cross the upper gastrointestinal absorption channel. Dietary proteins containing antioxidant peptide segments may explain their health benefits beyond regular nutrition. Human digestion primarily involves pepsin, an endopeptidase with strong cleavage activity for peptide bonds linked to hydrophobic amino acids, particularly phenylalanine, tyrosine, tryptophan, aromatic properties, and carboxyl-side amino acids like leucine and glutamic acid. In the small intestine, pancreatin, comprising exopeptidases (carboxypeptidases A and B) and endopeptidases (trypsin, α-chymotrypsin, and elastase), cleaves peptide bonds involving amino acids such as arginine and lysine due to its broad specificity [31].

The findings in this research indicate the importance of Phlai protein to provide bioactive peptides which are released when conditions approximating gastrointestinal digestion are replicated *in vitro*. Table 1 confirms that sequential incubation with pepsin and pancreatin produced potent digests with significant antioxidant activity. Hydrolyzed samples outperformed non-hydrolyzed ones in terms of antioxidant capacity. Enzymatic hydrolysis releases bioactive peptides with hydrogen donors that react with free radicals, terminating chain reactions. Antioxidant peptides in PPH obtained through pepsin-pancreatin digestion likely contain strong hydrophobic residues in their amino acid sequences, contributing to their potent antioxidant activity. In previous studies investigating the antioxidant activity of peptides obtained through pepsin-pancreatin hydrolysis, Saisavoey *et al*. [32] showed that pepsin hydrolysates derived from protein in rice bran could successfully scavenge free radicals. In addition, sunflower protein was hydrolyzed using pepsin and pancreatin by Megías *et al*. [33] who reported that the resulting hydrolysate demonstrated notable copper-chelating characteristics because the peptide sequence contained histidine and arginine. Meanwhile, Girgih *et al*. [34] hydrolyzed hemp protein isolate, initially with pepsin, and then with pancreatin in order to create biopeptides. It was possible to identify 23 peptide sequences from this particular protein hydrolysate, whereupon *in vitro* and *in vivo* testing showed that the identified peptides offered excellent antioxidant properties.

**Table 1:** Summary of values for ABTS, DPPH, NO, and FRAP indicating the IC_50_ values, for crude protein, PPH, and the five PPH-fractions derived through pepsin-pancreatin hydrolysis (using 10 kDa, 5 kDa, 3 kDa, and 0.65 kDa MWCO membranes).

MWCO fraction	Free radical scavenging activity (IC_50_) (μg/mL)			FRAP value (mM Fe^2+^/mg)
	ABTS	DPPH	NO	
crude extract	2.476±0.029^c^	0.811±0.021^d^	4.230±0.001^f^	2.592±0.012^d^
crude protein	2.528±0.122^c^	0.746±0.013^c^	2.220±0.033^c^	3.449±0.046^e^
protein hydrolysate	3.357±0.077^e^	1.267±0.008^e^	5.710±0.113^g^	1.701±0.039^c^
5–10 kDa	2.866±0.044^d^	2.674±0.023^g^	2.670±0.017^d^	0.989±0.006^b^
3–5 kDa	3.670±0.056^f^	2.492±0.082^f^	3.893±0.021^e^	1.134±0.029^c^
0.65–3 kDa	0.629±0.003^b^	0.522±0.012^b^	1.077±0.002^b^	0.452±0.022^a^
<0.65 kDa	0.011±0.006^a^	0.019±0.004^a^	0.030±0.005^a^	0.435±0.014^a^

Data are expressed in the form of mean ± standard error and the results are generated in triplicate.

^a-f^Those values with different letters appearing in the same row show significant differences (p>0.05).

### Free radical scavenging activity after PPH size fractionation using ultrafiltration

It is important to produce peptides of smaller size since they can be beneficial for health. This can be done *via* ultrafiltration, whereby a membrane allows fractionation to create smaller sized peptides. This approach works better than traditional techniques, such as gel chromatography, due to the lower cost, scalability, efficiency, and energy savings. In addition, the approach is environmentally friendly and can be carried out at room temperature. As a result, there are no undesirable precipitates formed during the process of separation [35]. In this study, the pepsin-pancreatin PPH underwent fractionation *via* ultrafiltration using MWCO membranes of 0.65, 3, 5 and 10 kDa. The best radical scavenging potential according to the findings shown in Table 1 came from the smallest MWCO fraction (MW < 0.65 kDa), which was selected ahead of the other fractions for further examination. Peptides with low molecular weight typically offer strong antioxidant activity due to lower steric hindrance, while the hydrophilic and hydrophobic amino acid residues form the active components of short chains and are therefore readily available for reactions with free radicals. They can also act as carriers capable of being absorbed through the intestinal barrier, thus enabling them to perform their antioxidative role [36]. It has also been shown in the literature that the antioxidant activity of many fractions is enhanced *via* ultrafiltration, as confirmed by Aursuwanna *et al*. [17] who reported that hydrolysates drawn from the proteins of lingzhi mushrooms showed better scavenging ability in the case of the fractions of smaller molecular size. Similarly, Sonklin *et al* [37]. found that hydrolysates from the proteins of mungbean meal offered superior DPPH scavenging capabilities for the fractions smaller than 1 kDa, while Wu *et al*. [38] found higher scavenging ability for fractions of low molecular weight obtained from Douchi hydrolysates. It may be the case that the fractions of lower molecular size contain a larger proportion of hydrophobic amino acids when compared to bigger peptides, and these are able to interact with peroxyl DPPH radicals. Zou *et al*. [36] have argued that peptide size is crucial when considering antioxidant capability, with lower molecular mass associated with greater antioxidant activity. In addition, the DPPH radical scavenging activity performed by fractions of low molecular weight may also be affected by other factors such as the solubility of the peptides, which permits the peptides to bind to the free radicals much more easily than is the case for bigger peptides which do not dissolve so readily.

### RP-HPLC fractionation of MW < 0.65 kDa and assessment of the free radical scavenging activity

**Fig 1:**
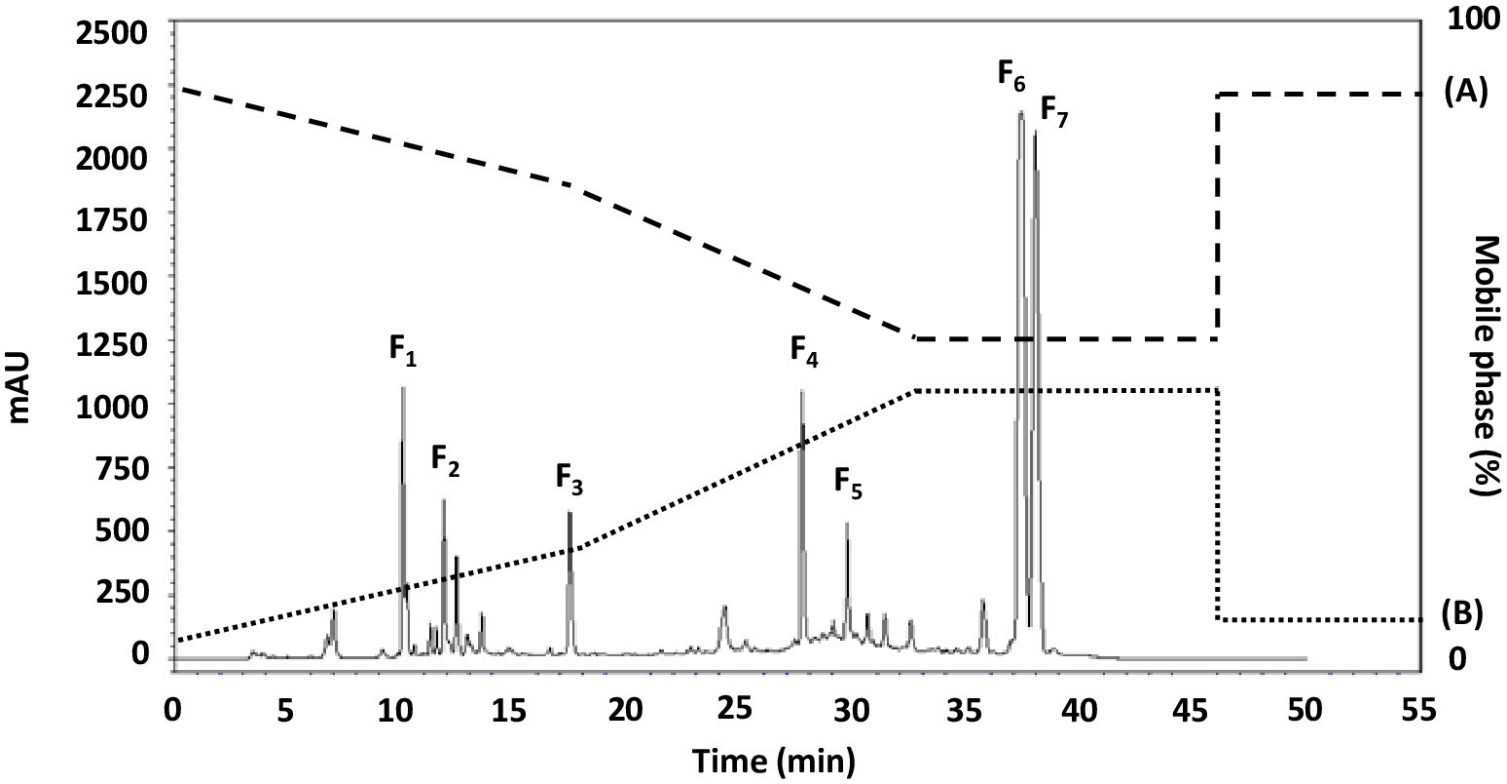
The RP-HPLC profile for the active fraction (< 0.65 kDa) from PPH.

The fractionation of PPHs in molecular weight lower than 0.65 kDa were used RP-HPLC technique with RP-C18 column and gradient elution while allow interaction with hydrophobic analyses within a polar mobile phase. Seven sub-fractions could be obtained as indicated in Fig 1. The fractions (F_1_-F_7_) were collected, lyophilized, concentrated, and examined antioxidant activity (ABTS, DPPH, NO, and FRAP), the sub-fraction (F_5_) showed only high potential antioxidant activity with IC_50_ values was 5.959 ± 0.204, 7.856 ± 0.040, 22.197 ± 5.254 mg/mL, and 0.759 ± 0.002 mM per Fe^2+^ mg, respectively. The yield of each purification procedure is shown in S2 Table. The F_5_ sub-fraction was then subjected to peptide amino acid sequencing through the use of Q-TOF-ESI-MS/MS analysis. According to the property of C_18_ column, the eluted order of each fraction peak was correlated to the polarity of samples. Generally, the hydrophobic components stay firmly within the column and are harder to be eluted out. The hydrophobic qualities and time taken are important to determine their anti-oxidative peptides that occur in chromatography column [39]. The longer time of elution can refer to high hydrophobicity and rise in anti-oxidative activity those depend on amino acid composition and peptides sequence in PPHs. It was previously reported for the peptides obtained from the protein hydrolysates of perilla seed protein hydrolysate, fraction c with the longest retention time and lowest molecular weight showed the highest DPPH radical-scavenging activity (87.7%) at a concentration of 0.5 mg/mL [40]. Meanwhile, Peng *et al*. [41] reported the elution of two peptides include bombinin-like peptide (BLP) and bombinin hydrophobic-type peptide (BH) were 92 and 147 minutes, respectively. These two peptides were able to act biologically against both microorganism and cancer because of that effect from their sequence and α-helical cationic structure, the capabilities for anticancer was demonstrated in BH peptide due to its greater hydrophobicity and high antioxidant activity.

### Identification of peptide sequences and prediction of bioinformatic properties

**Fig 2:**
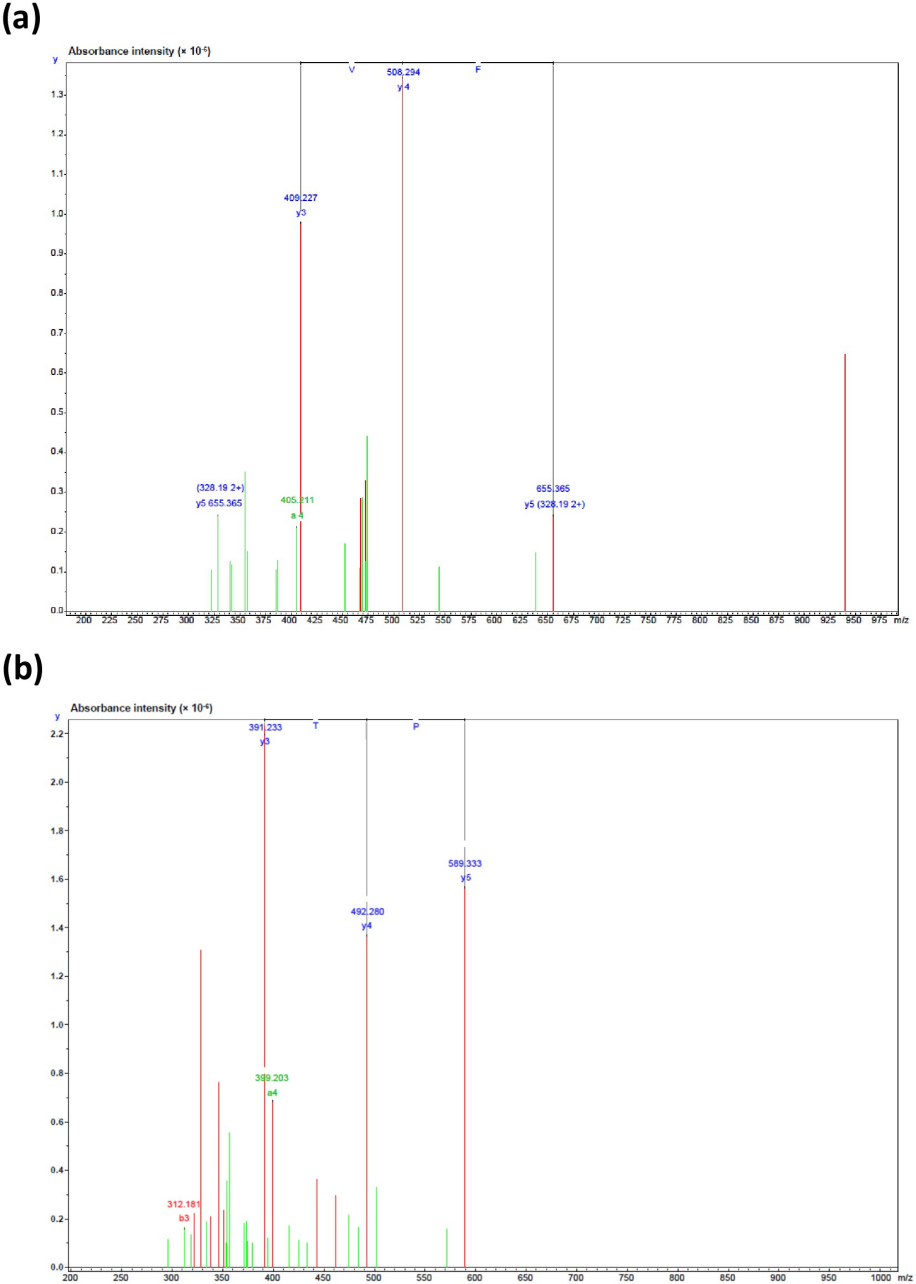
The mass fragmentation spectrum for the F_5_ sub-fraction obtained via reversed-phase high-performance liquid chromatography (see Fig 1): (a) DY-8, and (b) IK-6.

Following RP-HPLC, the best antioxidant F_5_ sub-fraction from PPH was selected and the amino acid sequence was determined *via* ESI-Q-TOF-MS/MS analysis. The mass spectra (MS) of the antioxidant peptides obtained from this F_5_ sub-fraction can be seen in Fig 2. The mass spectral analysis presented evidence of a number of previously unreported peptides: (1) Asp-Gly-Ile-Phe-Val-Leu-Asn-Tyr (DGIFVLNY or DY-8; m/z = 940.478), and (2) Ile-Pro-Thr-Asp-Glu-Lys (IPTDEK or IK-6; m/z = 702.367). Alignment of the fragments using the SwissProt database (*de novo* deducing) was then conducted to determine the homologous region. In the case of DY-8, 100% (8/8) similarity was revealed for the amino acid sequence with the cytosolic endo-β-N-acetylglucosaminidase 1-like from *Z*. *officinale* (SwissProt accession number XP_042429169.1), along with hypothetical protein ZIOFF_004822 from *Z*. *officinale* (SwissProt accession number KAG6531052.1) which can be seen in S3 Table. IK-6 was shown to be similar to a hypothetical protein ZIOFF_061232, whereas the identity of the amino acid sequence provided a 100% (6/6) match with *Z*. *officinale* (SwissProt accession number KAG6477800.1), indicating that the protein could potentially act as a signaling or transport protein, as evidenced in S4 Table. However, the peptide is very short, and it is therefore difficult to find exact conflict sites, so the peptide could be replaced in the polymorphic regions of proteins derived from divergent subunit proteins. Innovagen’s server data affirms poor water solubility, as shown in Table 2, but our research indicates DY-8 boasts good water solubility below 5.0 mg/mL. Solubility declined with increasing concentration. DY-8’s hydrophobicity measured 50%, while IK-6 registered at 33.33%. Hydrophobicity, alongside charge and molecular size, influences peptide bioavailability in vitro. Xie *et al*. [42] explored the link between casein peptide hydrophobicity and *in vitro* bioavailability, revealing highly hydrophobic peptides exhibit strong bioavailability. However, it’s noted that excessive hydrophobicity can hinder in vitro bioavailability. Thus, while hydrophobicity is vital for transcellular transport, moderation is key, considering peptidase hydrolysis during absorption in *in vitro* peptide bioavailability assessment.

**Table 2:** Peptide property profiles for the DY-8 and IK-6 peptides.

List of properties	Peptide properties	
	DY-8	IK-6
Water solubility^a^	Poor	Good
Hydrophobicity (%)^b^	50%	33.33%
Possible biological activities^c^	IF, GI, DG, NY, LN, LNY (ACE inhibitor) VL (Glucose uptake stimulating peptide) GI, LN, NY, VL (DPP-IV inhibitor)	IP, PT, EK (ACE inhibitor) IP, PT, EK, TD (DPP-IV inhibitor)
Toxicity (SVM score)^d^	Non-Toxic (-1.15)	Non-Toxic (-0.84)
Allergenicity prediction^e^	Probable non-allergen	Probable non-allergen

^a^Innovagen server, can assist in assessing peptide solubility (www.innovagen.com/proteomics-tools).

^b^Calculated with the peptide property calculator (www.peptide2.com).

^c^Data provided by the BIOPEP database.

^d^Toxicity analysis of the peptides can be performed using the ToxinPred server (http://crdd.osdd.net/raghava/toxinpred/).

^e^Allergenic determinants by AllerTOP (http://www.ddg-pharmfac.net/AllerTOP/).

Where peptides are to be used in functional foods, it is necessary to screen for undesirable qualities such as toxicity and allergenicity. This can best be achieved using bioinformatics. This is important because the use of peptides depends upon safety as well as bioavailability, so virtual screening is a quick and cost-effective way to check for safety concerns such as toxicity. ToxinPred, a tool utilizing information on amino acid composition and location, supports in silico predictions for peptide toxicity. Machine learning models, aided by a quantitative matrix, predict toxicity based on known properties of the peptides [43]. The SVM scores in Table 2, falling below zero, affirm the non-toxic nature of the peptides. Simultaneously, the rising concern of food-related allergies affecting approximately 8% of children and up to 2% of adults underscores the need for proactive measures. As many common allergens are present in foods derived from plants or animals, the European Food Safety Authority (EFSA) advocates *in silico* testing to predict potential issues stemming from exposure to food proteins. AllerTOP, employing a k-nearest neighbor algorithm, classifies peptides using a training set comprising well-known allergens and non-allergens from various species [44]. AllerTOP analysis assures minimal risk of allergenic problems with DY-8 and IK-6 peptides. As a means of assessing the other activities of peptides, the cheapest and fastest method is to involve computer simulations, such as BIOPEP, [27] although it must be acknowledged that these results may be somewhat limited in terms of the biological activities the tool might consider. In the case of DY-8 and IK-6, it was indicated that these peptides should be bioactive, with BIOPEP indicating the likely inhibition of dipeptidyl peptidase-IV (DPP-IV) and angiotensin-converting enzyme I (ACE), in addition to activity which would stimulate the uptake of glucose. These predications have their basis in specific short sequences, primarily comprising just two or three amino acid residues, which are a part of the sequences as shown in Table 2.

### Antioxidant activity confirmation and the structure-activity association in synthesized peptides

After identifying the two peptides, DY-8 and IK-6, they underwent synthesis and testing to assess their antioxidant properties, as detailed in Table 3. In this study, we chose peptide synthesis post-isolation to ensure precision and reproducibility. Isolation from natural sources often yields complex mixtures, while synthesis provides well-defined and purified peptides with precise sequences. This approach enhances control, facilitates targeted investigations, and improves result reliability. Both peptides share sequences containing hydrophobic amino acids like Pro and Tyr, known for promoting antioxidant activity. The aromatic rings of Phe, Trp, and Tyr, play a role in pro-oxidant metal ion chelation, contributing to the observed antioxidant activity. Sequences with N-terminal hydrophobic amino acids (Val or Leu) and specific residues within the sequence (Pro, His, or Tyr) exhibit robust antioxidant activity. Additionally, a C-terminal with hydrophilic (Asn, Gln, Ser, and Thr) and polar residues (Arg, Asp, Glu, His, and Lys) further enhances antioxidant potential. Hydrogen-donating residues near the C-terminal support chelation and free radical scavenging, heightening antioxidant activity. Among the better residues for antioxidant activity, Trp and Tyr have been claimed to be excellent because of their hydrogen-donating indole groups, in the case of Trp, and phenolic groups in the case of Tyr. If these amino acids were located at the N- and C-terminus positions, the performance would be even better [45]. The 3D structures of the identified peptides, predicted using the PEP-FOLD tool V3.5 (S1 Fig), revealed diverse spatial structures and folding patterns, predominantly featuring β-turns (75%) or α-helices (60%) instead of β-sheets or random coils. The α-helix plays a significant role in peptide antioxidant activity due to its contextual constraints [46]. The DY-8 and IK-6 peptides exhibited α-helix and random coil structures, with no observed β-sheets. However, since peptides with good antioxidant activity typically have low molecular weight, the secondary structural factors might not exert a particularly powerful influence upon antioxidant activity.

**Table 3:** Free radical scavenging activities for the DY-8 and IK-6 peptides.

Synthesized peptides	Free radical scavenging activity (IC_50_) (μg/mL)			FRAP value (mM Fe^2+^/mg)
	ABTS	DPPH	NO	
DY-8	0.214±0.057^b^	11.343±0.023^a^	0.529±0.026^a^	0.0687±0.021^b^
IK-6	0.181±0.034^a^	14.917±0.036^b^	7.58±0.077^b^	0.0223±0.013^a^

Data are expressed in the form of mean ± standard error and the results are generated in triplicate.

^a,b^Mean values indicated by the same letter in superscript within the same row or column are not statistically significant (*p* > 0.05).

The assessment of radical scavenging activity through DPPH and ABTS revealed distinctions due to radical solubility in varied solvents. Alcoholic solvents dissolve DPPH, enabling it to accept electrons and hydrogen ions, while ABTS dissolves in organic media and water. This allows antioxidant assays with DPPH and ABTS to explore compounds with both lipophilic and hydrophilic characteristics [47]. In contrast, NO reacts with oxygen, generating nitrite and proxy nitrite, leading to inflammation and disease. The FRAP assay evaluates the reduction of ferric ions to ferrous ions in the presence of antioxidants [48]. DY-8 and IK-6 donate electrons, aiding in the reduction of ferric ions, correlating with their scavenging abilities in DPPH and ABTS assays. Results indicate that in aqueous solutions, DGIFVKNY exhibited lower scavenging activity, while IK-6 showed greater activity in the ABTS assay than in alcoholic solvents. This suggests that the peptides’ conformation in aqueous solutions enhances electron and hydrogen acceptance to eliminate free radicals. Additionally, IK-6’s smaller size may contribute to its superior antioxidant properties compared to DGIFVKNY.

Antioxidant peptides can have their abilities enhanced by hydrophobic amino acids, which lead to improvements in the interactions of peptides which can act against cancer. These peptides enter target organs *via* hydrophobic interactions with membrane lipid bilayers, scavenging radicals in the outer leaflets of tumor cell membranes, effectively targeting cancer cells. Sequences with charged (Glu), heterocyclic (Pro), and aromatic amino acids (Trp, Tyr, Phe) exhibit enhanced anticancer properties. Sae-Leaw *et al*. [49] reported that the gelatin peptides obtained from seabass skins contained hydrophobic amino acids which would improve the antioxidant activity due to the hydrophobic cellular targets, such as polyunsaturated chains of fatty acids, becoming more readily accessible to the peptides. Furthermore, Chalamaiah *et al*. [50] stated that low molecular weight peptides exhibited greater anticancer activity, since this increases the quantity of hydrophobic amino acids. Understanding the structural properties of peptides from food sources is crucial in deciphering anticancer potential due to differences in cytotoxicity linked to molecular weight.

### Cytotoxicity and CAA of DY-8 and IK-6 peptides on Caco-2 cells

**Fig 3:**
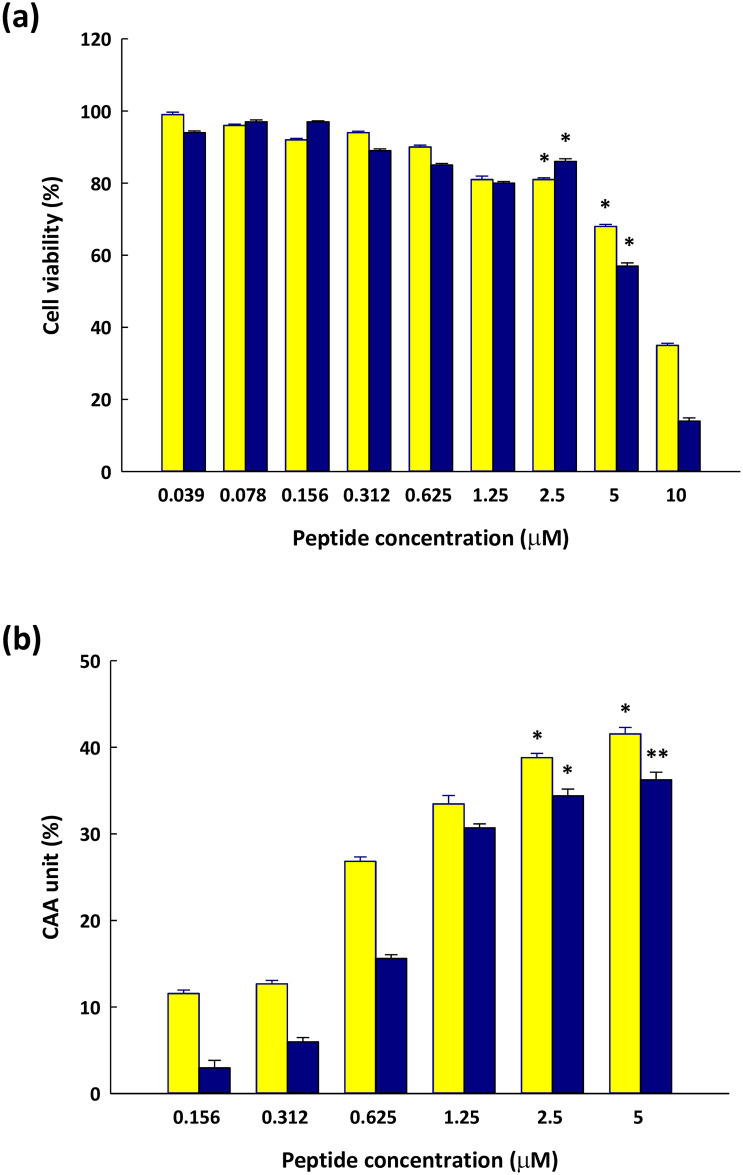
(a) Caco-2 cell viability after treatment at different concentrations of (yellow circle) DY-8 and (black circle) IK-6 for 72 hours, and (b) the cellular antioxidant activity of (yellow circle) DY-8 and (black circle) IK-6. Bars are used to indicate the standard deviation (n = 3).

The cytotoxicity analysis determined that the peptides DY-8 and IK-6 were both non-toxic at concentrations below 5 μM (with cell viability greater than 50%, as determined by MTT assay), as shown in Fig 3(a). Subsequent analysis revealed that both peptides demonstrated toxicity levels that exceeded 5 μM. No cytotoxic effects were detected below this threshold, suggesting a secure range for cellular viability. Specifically, cell survival was compromised at concentrations exceeding 5 μM, indicating the onset of toxicity. In the following experiments, concentrations of 0.156, 0.312, 0.625, 1.25, 2.5, and 5 μM were therefore selected. The antioxidant properties of DY-8 and IK-6 were next assessed with a popular *in vitro* oxidative stress model involving Caco-2 cells. This study used DCFH as a fluorescent probe to measure intracellular ROS production. The hydrolyzed DCFH-DA probe indicates oxidative stress in cells by transforming into a highly fluorescent DCF in the presence of ROS. Digesting phosvitin phosphopeptide under simulated gastrointestinal conditions decreased pro-inflammatory interleukin secretion in Caco-2 cells, suggesting its potential in improving gut health and preventing oxidative stress [51]. From these findings, it is likely that DY-8 and IK-6 would be able to prevent ROS from causing oxidative damage to cells, and therefore the peptides might in future be employed to prevent ROS formation. Where the DY-8 and IK-6 peptides were involved in pre-treatment, DCF fluorescent intensity declined significantly. Results in Fig 3(b) show that DCF fluorescent intensity was similar for the peptides DY-8 and IK-6 at every concentration up to 5 mM, suggesting a cellular radical scavenging effect from both peptides.

It is understood that the DY-8 and IK-6 peptides have hydrophobic and antioxidant amino acid residues, and these hydrophobic amino acid residues. Accordingly, it might be the case that these amino acid residues support DY-8 and IK-6 in controlling the oxidative damage caused by ROS. The production of ROS is widely considered to be cytotoxic, and has been linked to various adverse health conditions. As the concentration of ROS increases at the gastrointestinal level, intestinal pathologies are more likely to develop, so it would be useful to discover food antioxidants capable of protecting the intestinal epithelium when oxidative stress occurs. Both DY-8 and IK-6 have shown such abilities in maintaining cell viability and preventing intestinal oxidative damage when tested using the Caco-2 cells. Similar findings have been reported for the split gill [51] and lingzhi edible mushrooms, [17] which deliver protection in ABAP-stimulated Caco-2 cells. These compounds are known to be both healthy and safe, with a low molecular weight. Their activity levels are high and they can be readily absorbed. They also have the ability to combine well with non-peptide antioxidants to produce a better protective outcome.

### Analysis of flow cytometry

**Fig 4:**
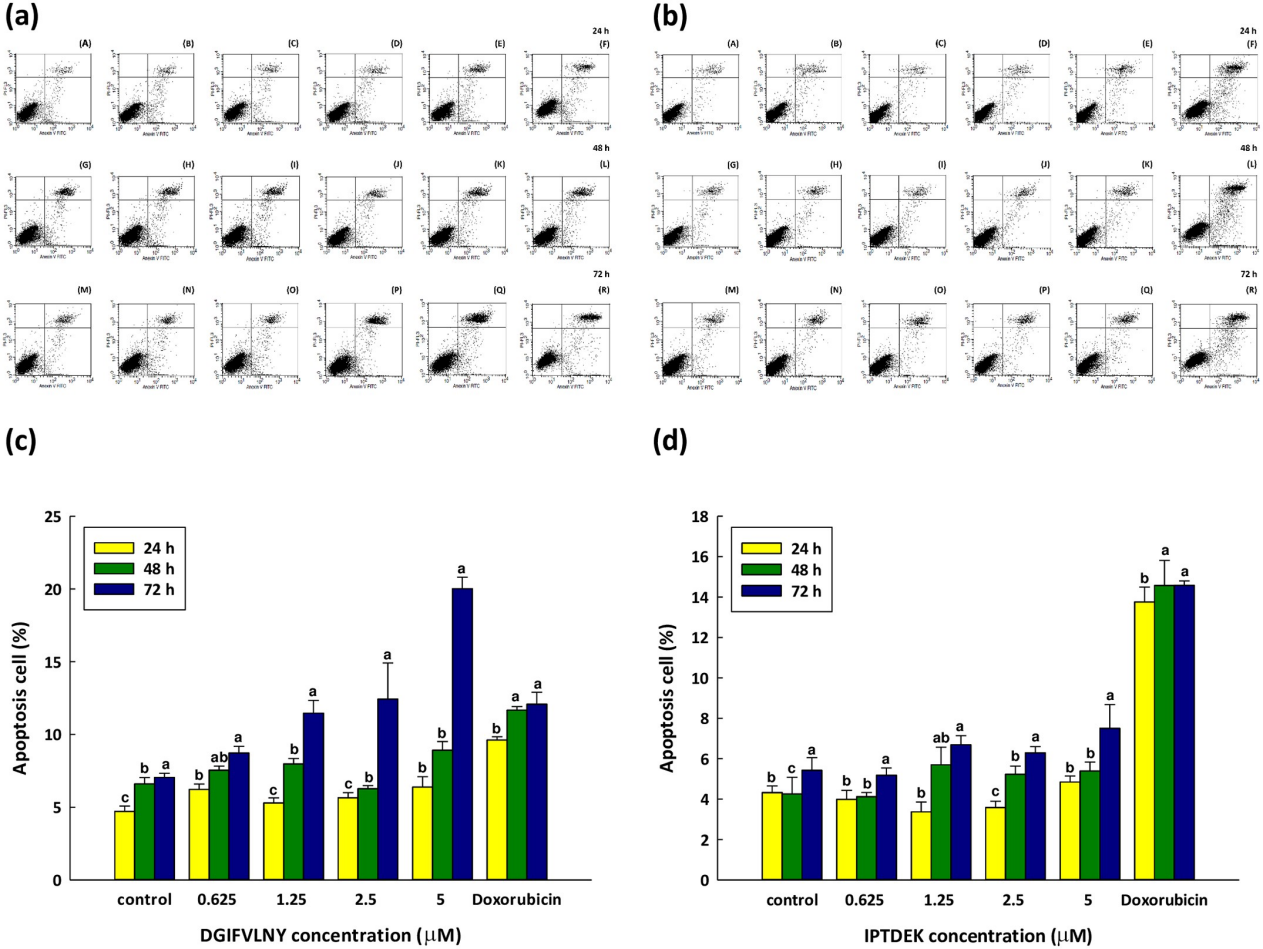
The flow cytometry analysis for Caco-2 cells which had been cultured for 24 hours (A–F), 48 hours (G–L), or 72 hours (M–R) in CM. Cells are labeled with FITC-annexin V and PI following culture with different (a) DY-8, and (b) IK-6 peptide concentrations. (A, G, and M) nothing (negative control), (B, H, and N) 0.625 μM, (C, I, O) 1.25 μM, (D, J, P) 2.5 μM, (E, K, Q) 5 μM of DY-8 peptide and (F, L, and R) 0.5 μg/mL doxorubicin (positive control). Quadrants: Bottom left—normal or live cells; Top left—necrotic cells; Bottom right—early apoptotic cells; Top right—late apoptotic cells. These data from 3 trials underwent conversion to Annexin V and viability dot plots. Bar graph presenting apoptotic cells; (c) DY-8, and (d) IK-6 peptide, each bar shows the mean ± standard deviation while all tests are carried out in triplicate. ^a-c^Values within a row with different letters are significantly different (p > 0.05).

To assess the apoptotic capacity of DY-8 and IK-6 peptides, varying concentrations and incubation periods were applied to Caco-2 cells. Apoptosis rates were determined using annexin V-FITC/PI double staining analysis, with concentrations below 5 μM chosen for assessment. Notably, while the peptides induced apoptosis, no significant cytotoxicity was observed, underscoring the controlled nature of apoptosis compared to general cell damage. These findings underscore the effectiveness of the selected peptide concentrations in inducing apoptosis without compromising overall cell viability. The results revealed a significant increase in apoptosis rates in Caco-2 cells, particularly with higher peptide concentrations and longer incubation durations. With an incubation period of 72 hours at different peptide concentrations, the apoptotic cell percentages shown in Fig 4 for the Caco-2 cells in the case of both the DY-8 peptide and also the IK-6 peptide, confirming that cell apoptosis induced by these peptides occurred in a dose-dependent manner. In earlier research, human cervical cancer cells were examined for cell apoptosis when exposed to the ZXR-1 peptide (128 μg/mL) for 6 hours. Early apoptosis results were 55.1%, compared to 2.47% for the control, while late apoptosis measured 5.8%, compared to 0.3% for the control. It was apparent that the ZXR-1 peptide was able to promote apoptosis through the targeting of the mitochondrial membrane which released apoptotic factors *via* the intrinsic pathways [52]. Similarly, the work of Ortiz-Martinez *et al*. [53] compared the antiproliferative effects of the peptide fractions obtained from albumin alcalase hydrolysates of white hybrid (Asgrow-773) and those obtained from quality protein (CML-502) maize. A model of human liver cancer was created *in vitro*, and it was found that the apoptosis rates of HepG2 cells exposed to Asgrow-773 were greater than was the case for CML-502 according to the flow cytometry results. In this experiment, the control represented 100% of the cells, whereupon every fold change indicated an increase by 100%. For Asgrow-773, the rise in late apoptotic events was evaluated as 484%, while for CML-502 the value was 400%. Furthermore, CML-502 saw a rise in early apoptotic events amounting to 567%. The findings indicated that both genotypes exerted antiproliferative effects by inducing the apoptosis of HepG2 cells.

### Apoptosis-related gene expression influenced by DY-8 and IK-6

**Fig 5:**
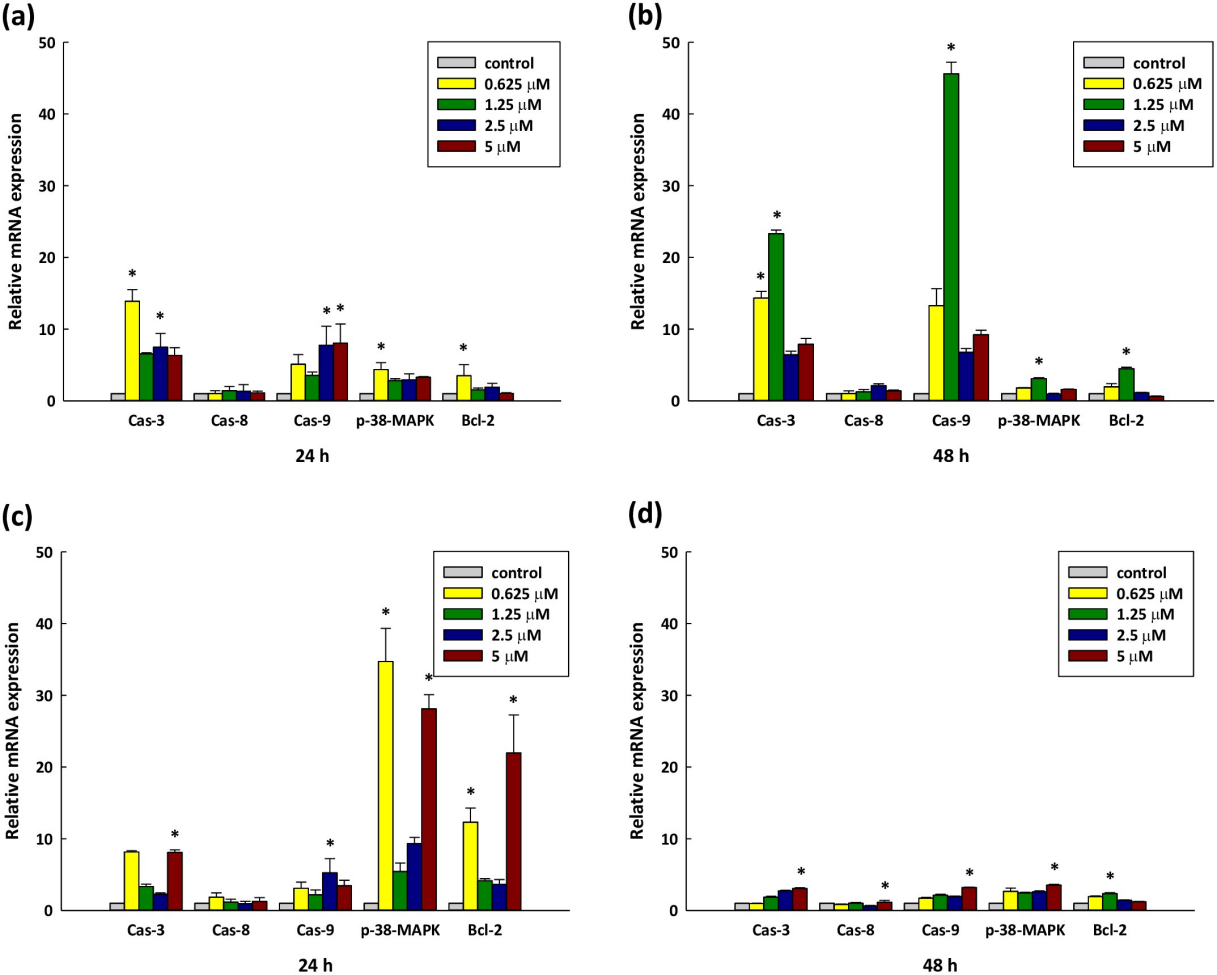
The influence of the DY-8 and IK-6 peptides upon apoptotic signaling pathways. Cells underwent incubation at different concentrations for 24 hours (A, C) with DY-8 peptide, and 48 hours (B, D) with IK-6 peptide. Following treatment, apoptotic mRNA transcripts were evaluated *via* quantitative reverse transcription—polymerase chain reaction (RT-qPCR). (mRNA expression normalization was performed against β-actin, to the control). Experiments were carried out in triplicate with 3 biological replicates. Each point represents the mean ± S.D. *p < 0.05 indicates a significant difference in comparison to the non-treated control *via* one-way ANOVA.

There are two caspase-dependent pathways which serve to mediate the morphological and biochemical changes in cells due to apoptosis. These are the extrinsic pathway, known as the death receptor, in which caspase-8 is activated, and the intrinsic, or mitochondrial, pathway in which caspase-9 activation occurs. In either scenario, the caspase activation results in the further activation of caspase-3. The caspases are intracellular cysteine proteases which show specificity for aspartic acid residues, and when triggered by drugs they can induce apoptosis in many different kinds of cancer cells. The initiator and receptor caspases have specific roles to play in apoptosis. In a majority of apoptotic pathways, caspase-3 cleaves and breaks down the different parts of the cells which are involved in DNA repair and regulation. After activation, caspase-3 cleaves various cellular substrates, which in turn causes membrane blebbing, the fragmentation of DNA, cell structure disassembly, and eventually the death of the cell [54]. To learn more about the pathways for cell apoptosis which would be induced by DY-8 and IK-6, this study evaluated the caspase-3, -8, -9, p38-MAPK, and Bcl-2 activity in the context of treated Caco-2 cells. Fig 5 shows that when Caco-2 cells are introduced to DY-8 or IK-6, the caspase-3 and -9 activities are significantly increased in comparison to untreated cells, with the response significantly affected by both time and dosage (p < 0.05). Caspase-3 expression was upregulated 5-fold, while for caspase-9 the upregulation was 11-fold, after 48 hours of treatment with 1.25 μM of the DY-8 peptide. In contrast, caspase-8 showed no notable changes with various concentrations or incubation periods in comparison to the control, and therefore it is apparent that the induced apoptosis may involve the intrinsic pathway in this particular cell line. Furthermore, when the IK-6 peptide is activated, this leads to a notable increase in p38-MAPK gene expression when compared to the situation with the DY-8 peptide. Apoptosis can be induced by p38-MAPK as it alters the mitochondrial membrane properties, causing cytochrome C to be released, and in turn affecting the caspase-3 and -9 expression. Meanwhile, the DY-8 peptide has the activation effect of increasing the expression of caspase-3 and -9 to a greater extent than occurs with the IK-6 peptide. This may be because higher levels of p38-MAPK expression do not lead to apoptosis only *via* the upregulation of caspase-3 and -9, but instead can have an influence on various cellular mechanisms, such as proliferation and proinflammatory cytokine release, including the release of tumor necrosis factor. Research conducted by Fan *et al*. [55] revealed the influence of three peptides derived from quinoa, finding that they were able to induce p38-MAPK-related gene expression, which strongly upregulated caspase-3 and -9 in the context of Caco-2 cells. Meanwhile, Rasaratnam *et al*. [56] studied a VS-9, a new anticancer peptide obtained from garlic, which could inhibit MOLT-4 and K562 leukemic cell line proliferation while simultaneously having almost no effect upon normal PBMC. VS-9 was able to induce apoptosis while upregulating the mRNA levels of caspase-3, -8, -9, and Bax, and downregulating Bcl-2, Bcl-xL, and Bcl-w. In the case of peptides obtained from *Laminaria japonica*, there was a rise in p38-MAPK and caspase-3-related gene expression when the peptides were introduced to H22 tumor cells [57].

### Influence on protein expression of DY-8 in the context of apoptosis

**Fig 6:**
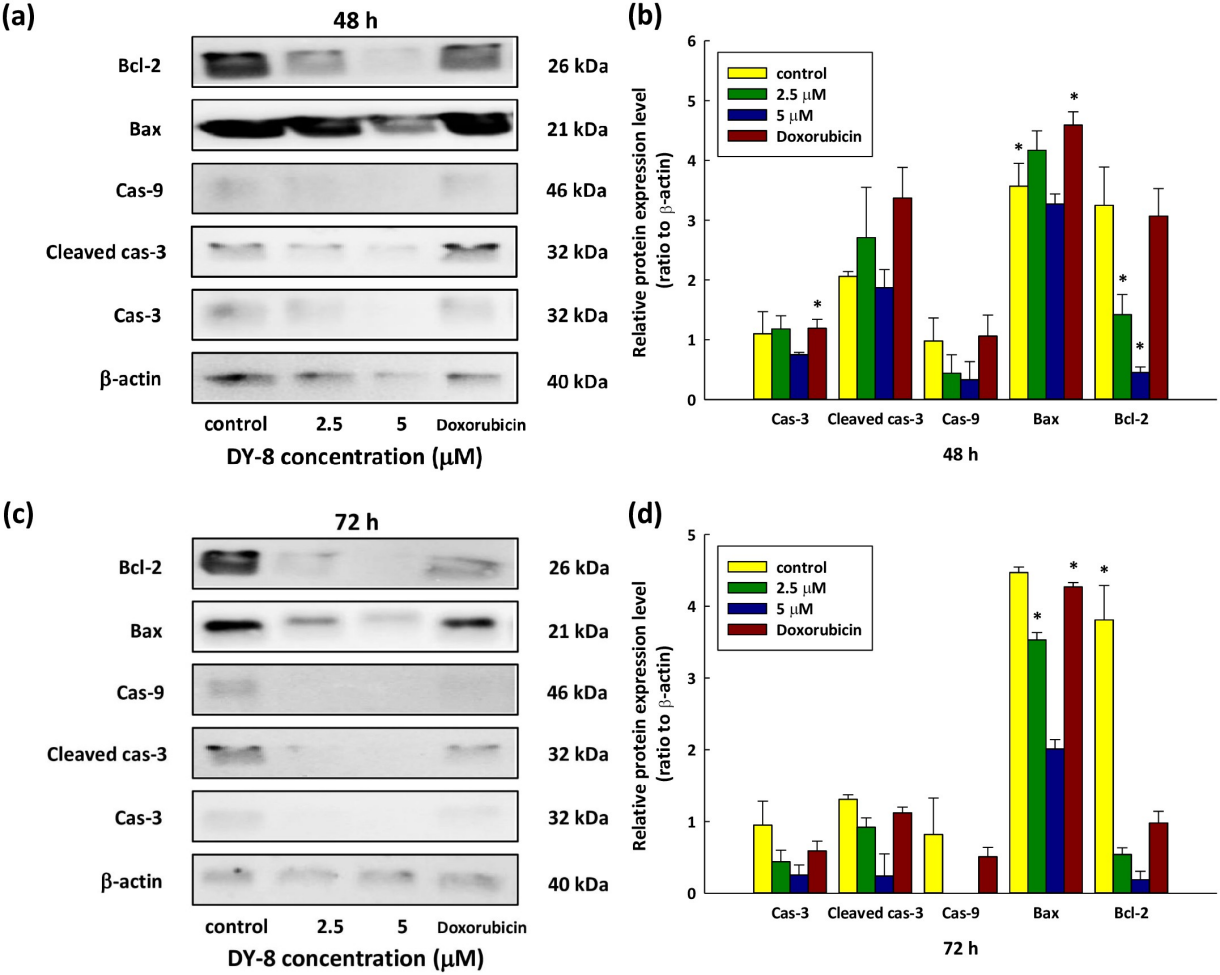
Western blot analyses indicate changes in the associated protein levels for the apoptotic Caco-2 cells following treatment with the DY-8 peptide. Whole-cell lysates prepared from Caco-2 cells underwent treatment using the DY-8 peptide at different concentrations for 48 hours (a, b) and 72 hours (c, d), followed by immunoblotting with anti-caspase-3, anti-cleaved caspase-3, anti-caspase-9, anti-Bax, anti-Bcl-2, and anti-β-actin antibodies. One representative blot drawn from 3 independent experiments is presented. The findings from independent experiments performed in triplicate can also be seen. Data take the form of mean ± S.D. of the fold changes (relative to β-actin expression). *p < 0.05 indicates a significant difference in comparison to the non-treated and DMSO-treated control *via* one-way ANOVA.

It was finally necessary to examine how DY-8 affects protein expression, and in turn how it can induce intrinsic apoptosis. The protein expression levels can be determined *via* Western blot analysis, investigating caspase-3, cleaved caspase-3, caspase-9, Bax, and Bcl-2, while β-actin was used as the loading control, as down in Fig 6 (S1 Raw images). Various concentrations were used, and the examination was carried out after 48 and 72 hours. Caspase-3 can indicate the induction of the mitochondrial apoptotic pathway, and its expression levels were shown to rise. For cleaved caspase-3, the induction of mitochondrial dysfunction was observed at a higher rate than the control, whereas Bcl-2, in contrast, is an anti-apoptotic protein whose expression levels dropped, suggesting that Bcl-2 played no role in disrupting mitochondrial functions such as cytochrome C release. Cell apoptosis is also supported by increased Bax expression. Bax is able to create a permeable membrane as it binds to the mitochondrial membrane, resulting in the formation of a mitochondrial membrane channel. Meanwhile, cytochrome C, which is a mitochondrial protein that serves to activate caspase-3, will signal intrinsic apoptosis [54]. There was an additional increase in caspase-9 expression along with the rise in caspase-3 expression, since it undergoes activation within the apoptosome complex in order to sustain catalytic status and also to activate caspase-3, leading to the induction of apoptosis at 48 hours (Fig 6(a) and 6(b)) and also 72 hours (Fig 6(c) and 6(d)) in the case of Caco-2 cells undergoing treatment with the DY-8 peptide. These findings show that the DY-8 peptide is able to induce apoptotic gene expression in Caco-2 cells, and that Bcl-2 and Bax proteins can regulate the apoptotic process by maintaining control over the mitochondrial function. The Bcl-2 monomers or homodimers play a role in promoting survival whereas Bax homodimers promote apoptosis. Furthermore, the activity of the ion channel relating to Bcl-2 and Bax might manage apoptosis through the effect upon mitochondrial membrane permeability. Bcl-2 and Bax both serve as transcriptional targets of p53, and when DNA damage occurs, this will result in the induction either of cell cycle arrest or apoptosis [54, 58]. This study suggests that p53 induces the up-regulation of Bax and down-regulation of Bcl-2 in Caco-2 cells treated with the DY-8 peptide.

From the molecular perspective, the intrinsic or the extrinsic apoptotic pathways activate the caspases. Mitochondrial membrane permeabilization is an important component of the intrinsic pathway, along with cytochrome C release into the cytosol, apoptosome formation, and the subsequent activation of caspase-9 and other downstream caspases, which can cause the fragmentation of DNA. The activation of executioner caspases such as caspase-3 and -7 can be modulated by caspase-9 *via* proteolysis, which transmits the apoptotic signal to the execution phase. Meanwhile, in the extrinsic pathway, procaspase-8 is an initiator caspase which undergoes proximity-induced auto-activation as it is recruited by the adaptor protein FADD (Fas-associated death domain) to the death domain-containing receptors [59]. The treatment of Caco-2 cells with the DY-8 peptide leads to the up-regulation of caspase-9 and -3, but there was no change to the level of caspase-8 mRNA. The findings from this current research indicate clearly that the DY-8 peptide is able to induce apoptosis effectively through the intrinsic mitochondrial pathway when treating Caco-2 cells. Earlier research has also found that bioactive peptides offer the possibility of inducing apoptosis-related protein expression. One bioactive peptide derived from the protein of chickpea seeds (*Cicer arietinum* L.) was able to induce apoptosis-related protein expression when used to treat the Ishikawa cell lines of human endometrial cancer. The outcome suggested lowered Bcl-2 expression and notably enhanced expression of caspase-3 and Bax in the Ishikawa cells according to the Western blot analysis results [60]. Meanwhile, the work of Fan *et al*. [55] showed that novel peptides obtained *via* the *in vitro* digestion of quinoa protein demonstrated anti-colon cancer activity when tested in Caco-2 cells. The Western blot findings revealed lower Bcl-2 expression but significantly higher caspase-3 expression. The progression of cancer might be arrested by quinoa peptides since they are able to regulate certain proteins related to cancer, so they may find a role in functional foods as a treatment for colon cancer. Finally, the work of Liang *et al*. [61] described the ability of rice-derived bran bioactive peptides (RBAP) to induce apoptosis-related proteins in human umbilical vein endothelial cells (HUVECs). The findings from the Western blot assay indicated the significant up-regulation of pro-caspase-3 and cleaved caspase-3 protein levels in the H_2_O_2_ injury group. However, the levels of pro-caspase-3 protein under H_2_O_2_ treatment underwent notable down-regulation in comparison to the control and to the group undergoing RBAP treatment. This suggests that the pro-caspase-3 and cleaved caspase-3 proteins rely upon the cytochrome C process and play important roles in cell apoptosis.

### System preparation and the molecular docking of antiapoptotic proteins

**Fig 7:**
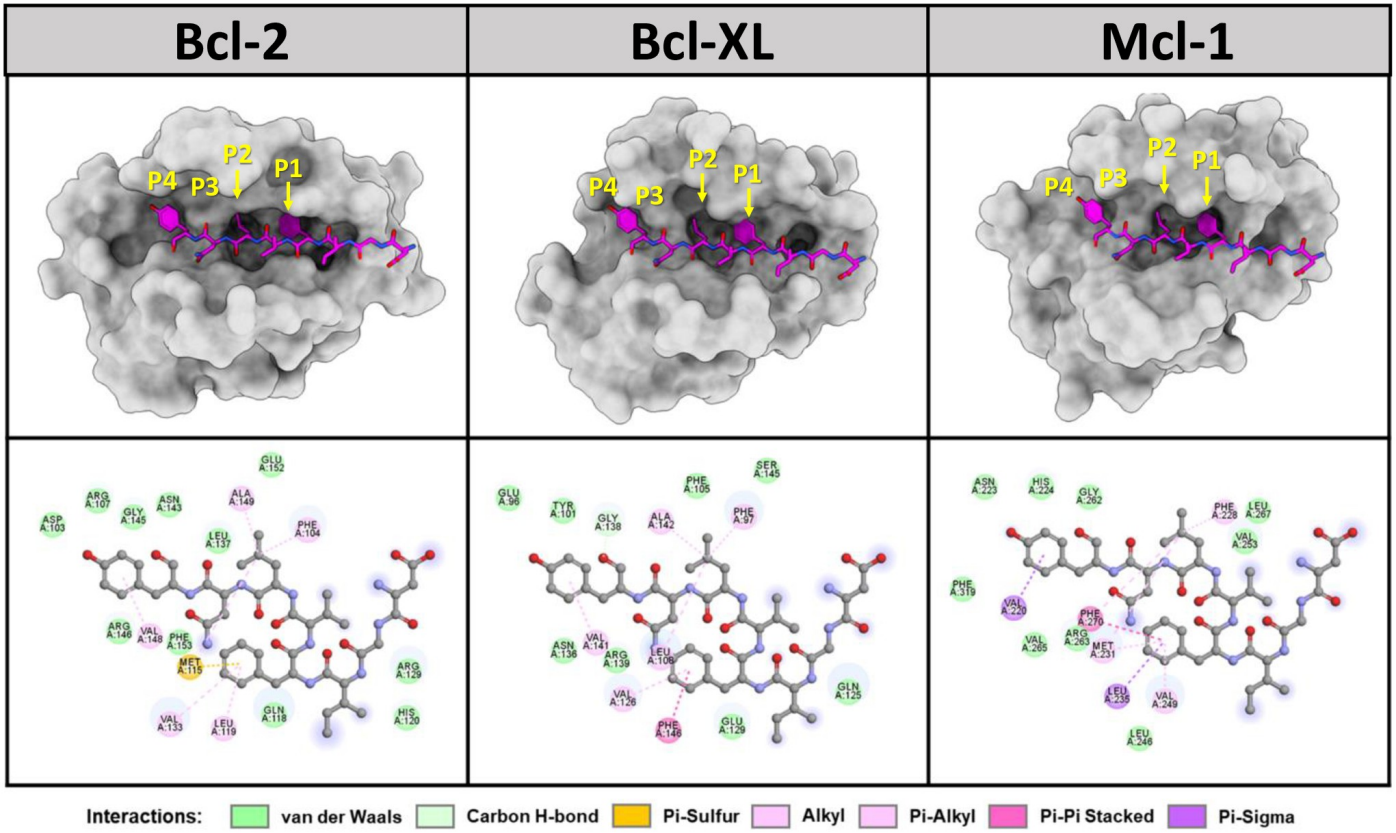
3D presentation of the molecular docking results (top) and 2D interactions (bottom) for the binding of the DY-8 peptide to three antiapoptotic proteins Bcl-2, Bcl-xL, and Mcl-1.

The HDOCK webserver was used to examine the molecular docking interactions of the DY-8 peptide with Bcl-2, Bcl-xL, and Mcl-1. Fig 7 shows that each of the antiapoptotic proteins under investigation had four hydrophobic groove pockets designated as P1-P4, whose role is to bind to the BH3 hydrophobic residues [62]. The findings confirm that F4 and L6 of the DY-8 peptide could be enclosed respectively by the P1 and P2 pockets. The stability of these two positions of the peptide was due to hydrophobic interactions with various protein residues: Met115, Leu119, and Val133 of Bcl-2, Val126 and Phe146 of Bcl-xL, as well as Met231, Leu235, Val249, and Phe270 of Mcl-1 toward F4, and also Phe104, Met115, and Ala149 of Bcl-2, Phe97, Leu108, and Ala142 of Bcl-xL, as well as Phe228, Met231, and Phe270 of Mcl-1 toward L6. This result concurs with the observation of the x-ray structure of the Bcl-2 antagonist, S55746, which binds to Bcl-2 to show that the phenyl moiety of S55746 was well occupied in the Bcl-2 P1 pocket [63, 64]. Notably, the hydrophobic residues (Leu94, Iso90, Iso97, and Phe101) of the Bim BH3 peptide exhibit intimacy with the hydrophobic residues in Bcl-xL, such as Leu, Val, Phe, and Tyr [65]. Bax BH3 peptide residues including Glu9, Ala13, Gly20, and Leu23 will bind to the sockets of Bcl-2 and Bax BH3 interactions [66]. Furthermore, Noxa and Puma form bonds to a hydrophobic groove in the Mcl-1 residues, such as Met212, Val230, Val234, Thr247, and Phe251 [67]. Similarly, the binding of the crystal structures of Bcl-xL and Mcl-1 to the BH3 peptide of BIM, and the binding of Bcl-2 to the Bax BH3 peptide indicate that these antiapoptotic proteins have a P2 pocket which is easily recognized by the amino acid leucine [25, 26, 68], just as the L6 of DY-8 peptide exhibited in our own docking results. In addition, it was possible for the amino acid tyrosine (Y8) at the peptide C-terminal to bind at the P4 pocket for each of the antiapoptotic proteins, creating van der Waals interactions with the adjacent residues as shown in Fig 7. Hydrophobic interactions were also observed between the Y8 residue and Val148 of Bcl-1, Val141 of Bcl-xL, and V220 of Mcl-1 *via* Pi-alkyl and Pi-sigma interactions. This result was appropriate based upon the structure of Bcl-xL and Mcl-1 in complex with the BIM BH3 peptide, suggesting that phenylalanine would be the ideal amino acid for binding to this pocket [24, 26, 68]. This result can be considered similar to the case in our own study of the aromatic amino acid tyrosine (Y8) of the DY-8 peptide. It is therefore the case that *in silico* results can provide valuable guidance in the synthesis of peptides which are better able to target the Bcl-2 family of antiapoptotic proteins.

## Conclusion

**Fig 8:**
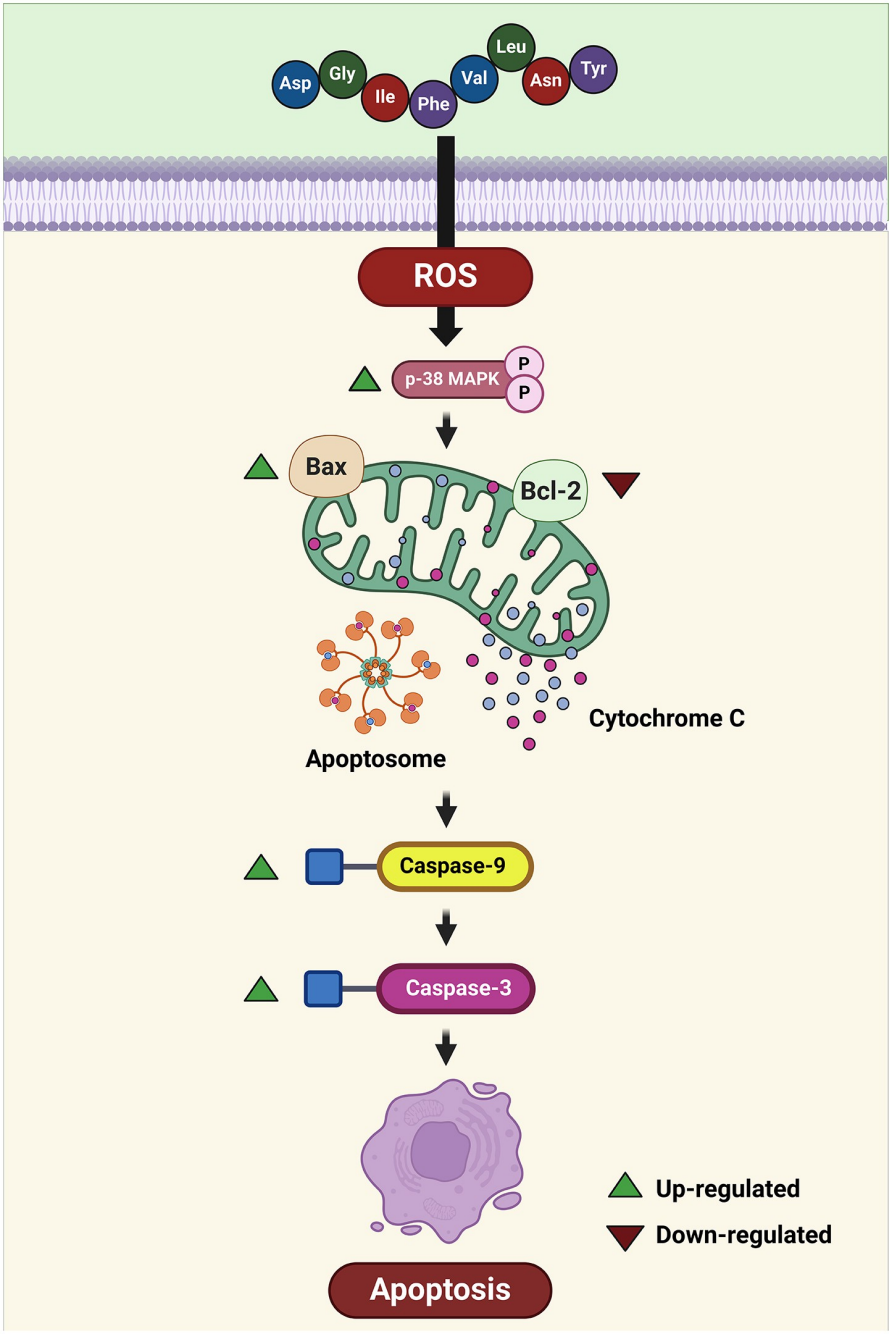
The hypothesized signaling network illustrates the intrinsic or mitochondrial apoptosis signaling pathway activated by the DY-8 peptide in the human colon cancer cell line (Caco-2). Bax: B-cell lymphoma protein 2 (Bcl-2)-associated X; Bcl-2: B-cell lymphoma-2; Caspase-3: cysteine protease-3; Caspase-9: cysteine protease-9; p38 MAPK: p38 mitogen-activated protein kinases; ROS: Reactive oxygen species. Biorender (^©^BioRender—biorender.com) was used to create the figure (S2 Fig).

This study demonstrated that hydrolysates from PPH using 2.5% (w/v) pepsin-pancreatin yield peptides with free radical scavenging abilities, confirmed by ABTS, DPPH, FRAP, and NO assays. Following membrane-separated ultrafiltration, low molecular weight peptides (<0.65 kDa) exhibited potent antioxidant activity. RP-HPLC fractionation yielded sub-fractions (F_1-7_), with F_5_ displaying superior activity in free radical scavenging. Amino acid sequencing identified two peptides, DY-8 and IK-6, synthesized and validated for antioxidant and cytoprotective effects on Caco-2 cells. Further analysis predicted peptide toxicity, and allergenicity. The DY-8 peptide demonstrated antioxidant activity which could be linked to the effects of the 3D structural components such as the α-helix. The findings suggest that the DY-8 peptide can induce apoptosis in Caco-2 cells through the induction in the beginning of the mitochondrial apoptotic pathway, whereupon the process gathers strength as the induction of intrinsic apoptosis signaling takes place as shown in Fig 8. The analysis of molecular docking suggests the optimal orientation and conformation for the DY-8 peptide in the context of the Bcl-2, Bcl-xL, and Mcl-1 protein binding sites in order to create a stable complex which can support the inhibitory reaction. It is clear from our results that the DY-8 peptide can potentially serve as an inhibitor of the targeted Bcl-2, Bcl-xL, and Mcl-1 proteins, on the basis of the high binding affinities reported. Accordingly, DY-8 should be studied further to examine its potential in an antitumor capacity. To develop effective therapies, it is important to better understand the molecular processes which take place, and also to focus upon safety issues for the benefit of patients in the future.

## Supporting information

S1 Fig 3D structure prediction of the DY-8, and IK-6 peptides. Each figures shows the 3D modeling of the identified peptide molecule, while the attached figures show the folding pattern in the aqueous solution of the molecule. Atomic code in the master figure.: red: oxygen, blue: nitrogen, gray: carbons, and white: hydrogen. Color code in the attached figure: red: α-helix, and gray: random coil.
(PDF)

S2 Fig Confirmation of publication and licensing rights. (PDF)

S1 Table Primer sequences and expected product sizes under quantitative PCR analysis. (PDF)

S2 Table Yield of each purification procedure. (PDF)

S3 Table Amino acid alignment of the DY-8 peptides in the homologous region as determined by protein BLAST. (PDF)

S4 Table Amino acid alignment of the IK-6 peptides in the homologous region as determined by protein BLAST. (PDF)

S1 Text List of used primary antibodies and the sources. β-actin (rabbit polyclonal; 1: 500 dilution), anti-Bcl-2 (mouse monoclonal; 1: 500 dilution), anti-Bax (mouse monoclonal; 1: 1,000 dilution), anti-Caspase-3 (mouse monoclonal; 1: 500 dilution), anti-Cleavaged aspase-3 (mouse monoclonal; 1: 500 dilution), and anti-Caspase-9 (rabbit polyclonal; 1: 500 dilution).
(PDF)

S1 Raw images Original blot of protein expression of Bcl-2, Bax, caspase-9, cleaved caspase-3, caspase-3, and β-actin. L: protein ladder, C: untreated cells, D: 0.5 μg/mL doxorubicin and DY-8 peptide concentration at 2.5 (P_1_), and 5 (P_2_) μM.
(PDF)

## References

[1] Bray F, Ferlay J, Soerjomataram I, Siegel RL, Torre LA, Jemal A. Global cancer statistics 2018: GLOBOCAN estimates of incidence and mortality worldwide for 36 cancers in 185 countries. CA: A Cancer Journal for Clinicians. 2018; 68(6), 394–424. doi: 10.3322/caac.21492 30207593

[2] Tauffenberger A, Magistretti PJ. Reactive oxygen species: Beyond their reactive behavior. Neurochemical Research. 2021; 46: 77–87. doi: 10.1007/s11064-020-03208-7 33439432 PMC7829243

[3] Xie RH, Xiao S, Chen X, Wang B, Hu YY, Wang JH. Separation, enrichment and cytoprotection of antioxidant peptides from Xuanwei ham using aqueous two-phase extraction. 2024; Food Chemistry. 138600. doi: 10.1016/j.foodchem.2024.138600 38452500

[4] Zhang Y, Li Y, Quan Z, Xiao P, Duan JA. New Insights into Antioxidant Peptides: An Overview of Efficient Screening, Evaluation Models, Molecular Mechanisms, and Applications. Antioxidants. 2024; 13(2), 203. doi: 10.3390/antiox13020203 38397801 PMC10886007

[5] Faraji N, Arab SS, Doustmohammadi A, Daly NL, Khosroushahi AY. ApInAPDB: a database of apoptosis-inducing anticancer peptides. Scientific Reports. 2022; 12(1), 21341. doi: 10.1038/s41598-022-25530-6 36494486 PMC9734560

[6] Xu F, Zhang J, Wang Z, Yao Y, Atungulu GG, Ju X, et al. Absorption and metabolism of peptide WDHHAPQLR derived from rapeseed protein and inhibition of HUVEC apoptosis under oxidative stress. Journal of Agricultural and Food Chemistry. 2018; 66(20), 5178–5189. doi: 10.1021/acs.jafc.8b01620 29732892

[7] Zhang X, Feng C, Wang S, Wang Y, Fu Z, Zhang Y, et al. A novel amphibian-derived peptide alleviated ultraviolet B-induced photodamage in mice. Biomedicine & Pharmacotherapy. 2021; 136, 111258. doi: 10.1016/j.biopha.2021.111258 33482615

[8] Trinidad-Calderón PA, Varela-Chinchilla CD, García-Lara S. Natural peptides inducing cancer cell death: Mechanisms and properties of specific candidates for cancer therapeutics. Molecules. 2021; 26: 7453. doi: 10.3390/molecules26247453 34946535 PMC8708364

[9] Zhang Y, Wang C, Zhang W, Li X. Bioactive peptides for anticancer therapies. Biomaterials Translational. 2023; 4: 5–17. doi: 10.12336/biomatertransl.2023.01.003 37206303 PMC10189813

[10] Chongmelaxme B, Sruamsiri R, Dilokthornsakul P, Dhippayom T, Kongkaew C, Saokaew S, et al. Clinical effects of *Zingiber cassumunar* (Plai): A systematic review. Complementary Therapies in Medicine. 2017; 35: 70–77. doi: 10.1016/j.ctim.2017.09.009 29154071

[11] Rachkeeree A, Kantadoung K, Suksathan R, Puangpradab R, Page PA, Sommano SR. Nutritional compositions and phytochemical properties of the edible flowers from selected Zingiberaceae found in Thailand. Frontiers in Nutrition. 2018; 5: 3. doi: 10.3389/fnut.2018.00003 29450200 PMC5799243

[12] Han AR, Kim H, Piao D, Jung CH, Seo EK. Phytochemicals and bioactivities of *Zingiber cassumunar* Roxb. Molecules. 2021; 26: 2377. doi: 10.3390/molecules26082377 33921835 PMC8073654

[13] Parcheta M, Świsłocka R, Orzechowska S, Akimowicz M, Choińska R, Lewandowski W. Recent developments in effective antioxidants: The structure and antioxidant properties. Materials. 2021; 14(8): 1984. doi: 10.3390/ma14081984 33921014 PMC8071393

[14] Inthuwanarud K, Sangvanich P, Puthong S, Karnchanatat A. Antioxidant and antiproliferative activities of protein hydrolysate from the rhizomes of Zingiberaceae plants. Pakistan Journal of Pharmaceutical Sciences. 2016; 29: 1893–1900. . 28375103

[15] Petsantad P, Sangtanoo P, Srimongkol P, Saisavoey T, Reamtong O, Chaitanawisuti N, et al. The antioxidant potential of peptides obtained from the spotted babylon snail (*Babylonia areolata*) in treating human colon adenocarcinoma (Caco-2) cells. RSC Advances. 2020; 10: 25746–25757. doi: 10.1039/d0ra03261a 35518590 PMC9055304

[16] Bradford MM. A rapid and sensitive method for the quantitation of microgram quantities of protein utilizing the principle of protein-dye binding. Analytical Biochemistry. 1976; 72: 248–254. doi: 10.1006/abio.1976.9999 942051

[17] Aursuwanna T, Noitang S, Sangtanoo P, Srimongkol P, Saisavoey T, Puthong S, et al. Investigating the cellular antioxidant and anti-inflammatory effects of the novel peptides in lingzhi mushrooms. Heliyon. 2022; 8: e11067. doi: 10.1016/j.heliyon.2022.e11067 36303910 PMC9593296

[18] Saisavoey T, Sangtanoo P, Reamtong O, Karnchanatat A. Free radical scavenging and anti-inflammatory potential of a protein hydrolysate derived from salmon bones on RAW 264.7 macrophage cells. Journal of the Science of Food and Agriculture. 2019; 99: 5112–5121. doi: 10.1002/jsfa.9755 30982967

[19] Suttisuwan R, Phunpruch S, Saisavoey T, Sangtanoo P, Thongchul N, Karnchanatat A. Isolation and characterization of anti-inflammatory peptides derived from trypsin hydrolysis of microalgae protein (*Synechococcus* sp. VDW). Food Biotechnology. 2019; 33: 303–324. doi: 10.1080/08905436.2019.1673171 PMC6902293 31866749

[20] Benzie IF, Strain JJ. The ferric reducing ability of plasma (FRAP) as a measure of “antioxidant power”: the FRAP assay. Analytical Biochemistry. 1996; 239: 70–76. doi: 10.1006/abio.1996.0292 8660627

[21] Wolfe KL, Liu RH. Cellular antioxidant activity (CAA) assay for assessing antioxidants, foods, and dietary supplements. Journal of Agricultural and Food Chemistry. 2007; 55: 8896–8907. doi: 10.1021/jf0715166 17902627

[22] Livak KJ, Schmittgen TD. (2001). Analysis of relative gene expression data using real-time quantitative PCR and the 2^− ΔΔCT^ method. Methods. 2001; 25(4): 402–408. doi: 10.1006/meth.2001.1262 11846609

[23] Srimongkol P, Songserm P, Kuptawach K, Puthong S, Sangtanoo P, Thitiprasert S, et al. Sulfated polysaccharides derived from marine microalgae, *Synechococcus* sp. VDW, inhibit the human colon cancer cell line Caco-2 by promoting cell apoptosis via the JNK and p38 MAPK signaling pathway. Algal Research. 2023 69, 102919. doi: 10.1016/j.algal.2022.102919

[24] Ku B, Liang C, Jung JU, Oh BH. Evidence that inhibition of BAX activation by BCL-2 involves its tight and preferential interaction with the BH3 domain of BAX. Cell Research. 2011; 21: 627–641. doi: 10.1038/cr.2010.149 21060336 PMC3343310

[25] Lee EF, Sadowsky JD, Smith BJ, Czabotar PE, Peterson-Kaufman KJ, Colman PM, et al. High-resolution structural characterization of a helical alpha/beta-peptide foldamer bound to the anti-apoptotic protein Bcl-xL. Angewandte Chemie International Edition in English. 2009; 48: 4318–4322. doi: 10.1002/anie.200805761 19229915 PMC2843084

[26] Czabotar PE, Lee EF, van Delft MF, Day CL, Smith BJ, Huang DC, et al. Structural insights into the degradation of Mcl-1 induced by BH3 domains. Proceedings of the National Academy of Sciences. 2007; 104: 6217–6222. doi: 10.1073/pnas.0701297104 17389404 PMC1851040

[27] Salomon‐Ferrer R, Case DA, Walker RC. An overview of the Amber biomolecular simulation package. Wiley Interdisciplinary Reviews: Computational Molecular Science. 2013; 3: 198–210. doi: 10.1002/wcms.1121

[28] Yan Y, Tao H, He J, Huang SY. The HDOCK server for integrated protein-protein docking. Nature Protocols. 2020; 15: 1829–1852. doi: 10.1038/s41596-020-0312-x 32269383

[29] Pettersen EF, Goddard TD, Huang CC, Meng EC, Couch GS, Croll TI, et al. UCSF ChimeraX: Structure visualization for researchers, educators, and developers. Protein Science. 2021; 30: 70–82. doi: 10.1002/pro.3943 32881101 PMC7737788

[30] Mackie A, Mulet-Cabero AI, Torcello-Gómez A. Simulating human digestion: developing our knowledge to create healthier and more sustainable foods. Food & Function. 2020; 11: 9397–9431. doi: 10.1039/d0fo01981j 33107545

[31] Amigo L, Hernández-Ledesma B. Current evidence on the bioavailability of food bioactive peptides. Molecules. 2020; 25: 4479. doi: 10.3390/molecules25194479 33003506 PMC7582556

[32] Saisavoey T, Sangtanoo P, Reamtong O, Karnchanatat A. Antioxidant and anti-inflammatory effects of defatted rice bran (*Oryza sativa* L.) protein hydrolysates on RAW 264.7 macrophage cells. Journal of Food Biochemistry. 2016; 40: 731–40. doi: 10.1111/jfbc.12266

[33] Megías C, Pedroche J, Yust MM, Girón-Calle J, Alaiz M, Millán F, et al. Production of copper-chelating peptides after hydrolysis of sunflower proteins with pepsin and pancreatin. LWT—Food Science and Technology. 2008; 41: 1973–1977. doi: 10.1016/j.lwt.2007.11.010

[34] Girgih AT, He R, Malomo SA, Aluko RE. Structural and functional characterization of hemp seed (*Cannabis sativa* L.) protein-derived antioxidant and antihypertensive peptides. Journal of Functional Foods. 2014; 6: 384–394. doi: 10.1016/j.jff.2013.11.005

[35] Zhang Y, Liu L, Zhang M, Li S, Wu J, Sun Q, et al. The research progress of bioactive peptides derived from traditional natural products in China. Molecules. 2023; 28: 6421. doi: 10.3390/molecules28176421 37687249 PMC10489889

[36] Zou TB, He TP, Li HB, Tang HW, Xia EQ. The structure-activity relationship of the antioxidant peptides from natural proteins. Molecules. 2016; 21: 72. doi: 10.3390/molecules21010072 26771594 PMC6273900

[37] Sonklin C, Laohakunjit N, Kerdchoechuen O. Assessment of antioxidant properties of membrane ultrafiltration peptides from mungbean meal protein hydrolysates. PeerJ. 2018; 6: e5337. doi: 10.7717/peerj.5337 30065890 PMC6065462

[38] Wu L, Jiang A, Jing Y, Zheng Y, Yan Y. Antioxidant properties of protein hydrolysate from Douchi by membrane ultrafiltration. International Journal of Food Properties. 2017; 20: 997–1006. doi: 10.1080/10942912.2016.1192644

[39] Hu X, Yang X, Wu Q, Li L, Wu Y, Chen S, et al. Purification and identification of antioxidant peptides from *Schizochytrium Limacinum* hydrolysates by consecutive chromatography and electrospray ionization-mass spectrometry. Molecules. 2019; 24: 3004. doi: 10.3390/molecules24163004 31430953 PMC6719025

[40] Yang J, Hu L, Cai T, Chen Q, Ma Q, Yang J, et al. Purification and identification of two novel antioxidant peptides from perilla (*Perilla frutescens* L. Britton) seed protein hydrolysates. PLoS One. 2018; 13: e0200021. doi: 10.1371/journal.pone.0200021 29985955 PMC6037370

[41] Peng X, Zhou C, Hou X, Liu Y, Wang Z, Peng X, et al. Molecular characterization and bioactivity evaluation of two novel bombinin peptides from the skin secretion of oriental fire-bellied toad, *Bombina orientalis*. Amino Acids. 2018; 50: 241–253. doi: 10.1007/s00726-017-2509-z 29098406

[42] Xie N, Wang B, Jiang L, Liu C, Li B. Hydrophobicity exerts different effects on bioavailability and stability of antioxidant peptide fractions from casein during simulated gastrointestinal digestion and Caco-2 cell absorption. Food Research International. 2015; 76: 518–526. doi: 10.1016/j.foodres.2015.06.025 28455033

[43] Gupta S, Kapoor P, Chaudhary K, Gautam A, Kumar R; Open Source Drug Discovery Consortium; et al. *In silico* approach for predicting toxicity of peptides and proteins. *PLoS One*. 2013; 8: e73957. doi: 10.1371/journal.pone.0073957 24058508 PMC3772798

[44] Dimitrov I, Flower DR, Doytchinova I. AllerTOP v.2-a server for *in silico* prediction of allergens. Journal of Molecular Modeling. 2014; 20: 2278. doi: 10.1186/1471-2105-14-S6-S4 24878803

[45] Deng B, Long H, Tang T, Ni X, Chen J, Yang G, et al. Quantitative structure-activity relationship study of antioxidant tripeptides based on model population analysis. International Journal of Molecular Sciences. 2019; 20: 995. doi: 10.3390/ijms20040995 30823542 PMC6413046

[46] Kaur H, Garg A, Raghava GPS. PEPstr: A *de novo* method for tertiary structure prediction of small bioactive peptides. Protein &Peptide Letters. 2007; 14: 626–631. doi: 10.2174/092986607781483859 17897087

[47] Abramovič H, Grobin B, Poklar Ulrih N, Cigić B. Relevance and standardization of *in vitro* antioxidant assays: ABTS, DPPH, and folin-ciocalteu. Journal of Chemistry. 2018; doi: 10.1155/2018/4608405 .4608405

[48] Harsha SN, Anilakumar KR. *In vitro* free radical scavenging and DNA damage protective property of *Coriandrum sativum* L. leaves extract. Journal of Food Science and Technology. 2014; 51: 1533–1539. doi: 10.1007/s13197-012-0648-5 25114344 PMC4108673

[49] Sae-leaw T, O’Callaghan YC, Benjakul S, O’Brien NM. Antioxidant, immunomodulatory and antiproliferative effects of gelatin hydrolysates from seabass (*Lates calcarifer*) skins. International Journal of Food Science & Technology. 2016; 51: 1545–1551. doi: 10.1111/ijfs.13123 PMC4711425 26787942

[50] Chalamaiah M, Yu W, Wu J. Immunomodulatory and anticancer protein hydrolysates (peptides) from food proteins: A review. Food Chemistry. 2018; 245: 205–222. doi: 10.1016/j.foodchem.2017.10.087 29287362

[51] Wongaem A, Reamtong O, Srimongkol P, Sangtanoo P, Saisavoey T, Karnchanatat A. Antioxidant properties of peptides obtained from the split gill mushroom (*Schizophyllum commune*). Journal of Food Science and Technology. 2021; 58: 680–691. doi: 10.1007/s13197-020-04582-4 33568862 PMC7847919

[52] Zhou XR, Zhang Q, Tian XB, Cao YM, Liu ZQ, Fan R, et al. From a pro-apoptotic peptide to a lytic peptide: One single residue mutation. Biochimica et Biophysica Acta. 2016; 1858: 1914–1925. doi: 10.1016/j.bbamem.2016.05.012 27207743

[53] Ortiz-Martinez M, Gonzalez de Mejia E, García-Lara S, Aguilar O, Lopez-Castillo LM, Otero-Pappatheodorou JT. Antiproliferative effect of peptide fractions isolated from a quality protein maize, a white hybrid maize, and their derived peptides on hepatocarcinoma human HepG2 Cells. Journal of Functional Foods. 2017; 34: 36–48.

[54] Elmore S. Apoptosis: a review of programmed cell death. Toxicologic Pathology. 2007; 35: 495–516. doi: 10.1080/01926230701320337 17562483 PMC2117903

[55] Fan X, Guo H, Teng C, Zhang B, Blecker C, Ren G. Anti-colon cancer activity of novel peptides isolated from *in vitro* digestion of quinoa protein in caco-2 cells. Foods. 2022; 11: 194. doi: 10.3390/foods11020194 35053925 PMC8774364

[56] Rasaratnam K, Nantasenamat C, Phaonakrop N, Roytrakul S, Tanyong D. A novel peptide isolated from garlic shows anticancer effect against leukemic cell lines *via* interaction with Bcl-2 family proteins. Chemical Biology & Drug Design. 2021; 97: 1017–1028. doi: 10.1111/cbdd.13831 33595876

[57] Wu Y, Li Y, Guo W, Liu J, Lao W, Hu P, et al. *Laminaria japonica* peptides suppress liver cancer by inducing apoptosis: Possible signaling pathways and mechanism. Marine Drugs, 2022; 20: 704. doi: 10.3390/md20110704 36355026 PMC9698768

[58] Danial NN. BCL-2 family proteins: critical checkpoints of apoptotic cell death. Clinical Cancer Research. 2007; 13: 7254–7263. doi: 10.1158/1078-0432.CCR-07-1598 18094405

[59] Wu CC, Bratton SB. Regulation of the intrinsic apoptosis pathway by reactive oxygen species. Antioxidants & Redox Signaling 2013; 19: 546–558. doi: 10.1089/ars.2012.4905 22978471 PMC3717204

[60] Gupta N, Bhagyawant SS. Bioactive peptide *of Cicer arietinum* L. induces apoptosis in human endometrial cancer via DNA fragmentation and cell cycle arrest. 3 Biotech. 2021; 11: 63. doi: 10.1007/s13205-020-02614-6 33489681 PMC7803852

[61] Liang Y, Lin Q, Huang P, Wang Y, Li J, Zhang L, et al. Rice bioactive peptide binding with TLR4 to overcome H_2_O_2_-induced injury in human umbilical vein endothelial cells through NF-κB signaling. Journal of Agricultural and Food Chemistry. 2018; 66: 440–448. doi: 10.1021/acs.jafc.7b04036 29276944

[62] Adams JM, Cory S. The BCL-2 arbiters of apoptosis and their growing role as cancer targets. Cell Death & Differentiation. 2018; 25: 27–36. doi: 10.1038/cdd.2017.161 29099483 PMC5729526

[63] Casara P, Davidson J, Claperon A, Le Toumelin-Braizat G, Vogler M, Bruno A, et al. S55746 is a novel orally active BCL-2 selective and potent inhibitor that impairs hematological tumor growth. Oncotarget. 2018; 9: 20075. doi: 10.18632/oncotarget.24744 29732004 PMC5929447

[64] Birkinshaw RW, Gong JN, Luo CS, Lio D, White CA, Anderson MA, et al. Structures of BCL-2 in complex with venetoclax reveal the molecular basis of resistance mutations. Nature Communications. 2019; 10: 2385. doi: 10.1038/s41467-019-10363-1 31160589 PMC6547681

[65] Lama D, Sankararamakrishnan R. Anti-apoptotic Bcl-XL protein in complex with BH3 peptides of pro-apoptotic Bak, Bad, and Bim proteins: comparative molecular dynamics simulations. Proteins. 2008; 73: 492–514. doi: 10.1002/prot.22075 18452209

[66] Yi J, Kellner V, Joo H, Chien N, Patel S, Chaban Z, et al. Characterizing the consensus residue specificity and surface of BCL-2 binding to BH3 ligands using the Knob-Socket model. PLoS One. 2023; 18: 1–17. doi: 10.1371/journal.pone.0281463 36795726 PMC9934389

[67] Dalafave DS, Prisco G. Inhibition of antiapoptotic BCL-XL, BCL-2, and MCL-1 proteins by small molecule mimetics. Cancer Informatics. 2010; 9: 169–177. doi: 10.4137/cin.s5065 20838611 PMC2935820

[68] Fire E, Gullá SV, Grant RA, Keating AE. Mcl-1-Bim complexes accommodate surprising point mutations *via* minor structural changes. Protein Science. 2010; 19: 507–519. doi: 10.1002/pro.329 20066663 PMC2866276

