# Correction: A novel peptide derived from *Zingiber cassumunar* rhizomes exhibits anticancer activity against the colon adenocarcinoma cells (Caco-2) *via* the induction of intrinsic apoptosis signaling

**DOI:** 10.1371/journal.pone.0341235

**Published:** 2026-01-20

**Authors:** Kitjasit Promsut, Papassara Sangtanoo, Piroonporn Srimongkol, Tanatorn Saisavoey, Songchan Puthong, Anumart Buakeaw, Onrapak Reamtong, Bodee Nutho, Aphichart Karnchanatat

There are a number of errors in the caption for Fig 5, “The influence of the DY-8 and IK-6 peptides upon apoptotic signaling pathways”. Please see the complete, correct Fig 5 caption here.

**Fig 5:**
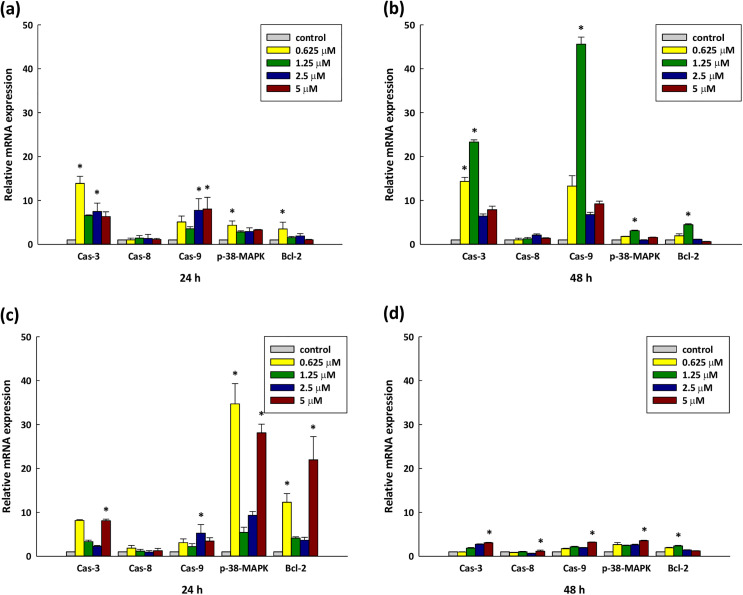
The influence of the DY-8 and IK-6 peptides upon apoptotic signaling pathways. Cells underwent incubation at different concentrations for 24 hours (A, C) and 48 hours (B, D) with DY-8 and IK-6 peptides, respectively. (A, B) represent DY-8 peptide treatment at 24 h and 48 h, while (C, D) represent IK-6 peptide treatment at 24 h and 48 h. Following treatment, apoptotic mRNA transcripts were evaluated via quantitative reverse transcription–polymerase chain reaction (RT-qPCR). (mRNA expression normalization was performed against β-actin, to the control). Experiments were carried out in triplicate with 3 biological replicates. Each point represents the mean ± S.D. *p < 0.05 indicates a significant difference in comparison to the non-treated control via one-way ANOVA.
