# Supplementary material for: A novel peptide derived from Zingiber cassumunar rhizomes exhibits anticancer activity against the colon adenocarcinoma cells (Caco-2) via the induction of intrinsic apoptosis signaling
Source: PLoS One. 2024 Jun 13;19(6):e0304701. doi: 10.1371/journal.pone.0304701 (PMC11175412; doi:10.1371/journal.pone.0304701)

S1 Fig. 3D structure prediction of the DY-8, and IK-6 peptides. Each figures shows the 3D modeling of the identified peptide molecule, while the attached figures show the folding pattern in the aqueous solution of the molecule. Atomic code in the master figure.: red: oxygen, blue: nitrogen, gray: carbons, and white: hydrogen. Color code in the attached figure: red: α-helix, and gray: random coil.

DY-8 peptide

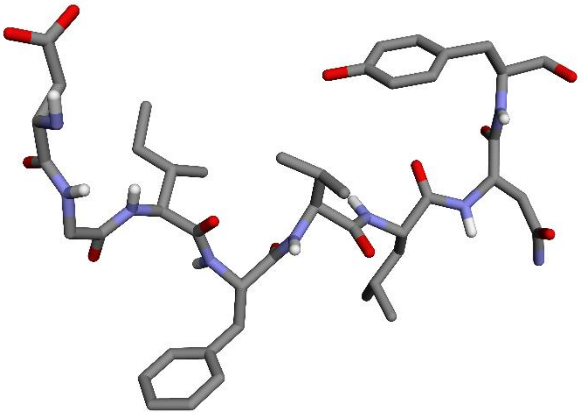

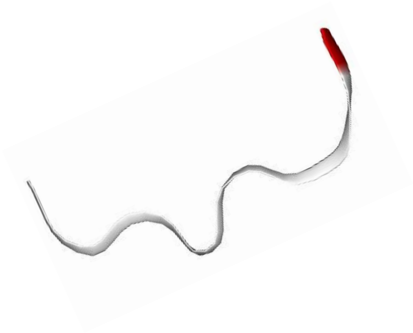

IK-6 peptide

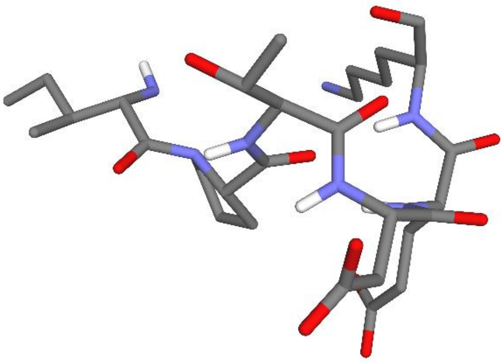

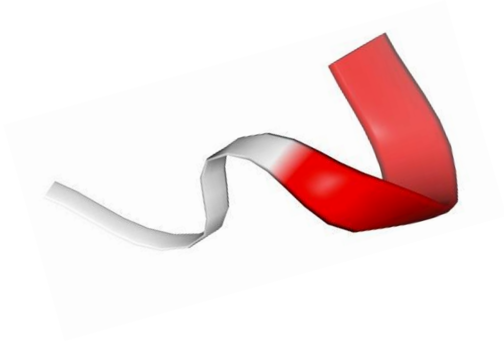

Supplement: S1 Fig — Each figures shows the 3D modeling of the identified peptide molecule, while the attached figures show the folding pattern in the aqueous solution of the molecule. Atomic code in the master figure.: red: oxygen, blue: nitrogen, gray: carbons, and white: hydrogen. Color code in the attached figure: red: α-helix, and gray: random coil. (PDF) [file pone.0304701.s001.pdf]
