# Supplementary material for: A novel peptide derived from Zingiber cassumunar rhizomes exhibits anticancer activity against the colon adenocarcinoma cells (Caco-2) via the induction of intrinsic apoptosis signaling
Source: PLoS One. 2024 Jun 13;19(6):e0304701. doi: 10.1371/journal.pone.0304701 (PMC11175412; doi:10.1371/journal.pone.0304701)
Supplement: S1 Table — (PDF) [file pone.0304701.s003.pdf]

S1 Table Primer sequences and expected product sizes under quantitative PCR analysis.

**Gene**	**Primer sequence (5′-3′)**	**Amplicon length**	**Tm (°C)**
β-actin	F: TCACCAACTGGGACGACATG	208	60
	F: TCACCAACTGGGACGACATG		
Caspase-3	F: TGCATACTCCACAGCACCTG	153	60
	R: TCTGTTGCCACCTTTCGGTT		
Caspase-8	F: CATCCAGTCACTTTGCCAGA	128	60
	R: GCATCTGTTTCCCCATGT TT		
Caspase-9	F: CTGAGGCAA GCCATAATC G	194	60
	R: AGAGGACATGGGAATAGCGT		
p-38 MAPK	F: CTGTTTCCCCAGAGCAGGAG	211	60
	R: GCTGGGTGCAGTACTTCCTT		
Bcl-2	F: GGGGTCATGTGTGTGGAGAG	158	60
	R: CCTCAGCCCAGACTCACATC		
