# Supplementary material for: A novel peptide derived from Zingiber cassumunar rhizomes exhibits anticancer activity against the colon adenocarcinoma cells (Caco-2) via the induction of intrinsic apoptosis signaling
Source: PLoS One. 2024 Jun 13;19(6):e0304701. doi: 10.1371/journal.pone.0304701 (PMC11175412; doi:10.1371/journal.pone.0304701)
Supplement: S2 Table — (PDF) [file pone.0304701.s004.pdf]

S2 Table Yield of each purification procedure

**Fractions**	**Yield (mg protein)**	**Yield (%)**
Crude extract	29.13 ± 0.35	100
Crude protein	26.41 ± 0.46	90.66
Crude protein hydrolysate	25.94 ± 0.76	89.04
Ultrafiltration		
MW 5-10 kDa fraction	15.84 ± 0.19	54.37
MW 3-5 kDa fraction	11.17 ± 0.07	38.35
MW 0.65-3 kDa fraction	5.66 ± 0.71	19.42
MW < 0.65 kDa fraction	1.58 ± 0.31	5.41
RP-HPLC		
F_1 sub-fraction	0.019 ± 0.06	0.065
F_2 sub-fraction	0.027 ± 0.08	0.093
F_3 sub-fraction	0.039 ± 0.06	0.134
F_4 sub-fraction	0.089 ± 0.02	0.306
F_5 sub-fraction	0.143 ± 0.01	0.491
F_6 sub-fraction	0.238 ± 0.02	0.817
F_7 sub-fraction	0.253 ± 0.01	0.869

All the data are given as the mean ± standard deviation of the triplicates.
