# Supplementary material for: A novel peptide derived from Zingiber cassumunar rhizomes exhibits anticancer activity against the colon adenocarcinoma cells (Caco-2) via the induction of intrinsic apoptosis signaling
Source: PLoS One. 2024 Jun 13;19(6):e0304701. doi: 10.1371/journal.pone.0304701 (PMC11175412; doi:10.1371/journal.pone.0304701)
Supplement: S3 Table — (PDF) [file pone.0304701.s005.pdf]

S3 Table Amino acid alignment of the DY-8 peptides in the homologous region as determined by Protein BLAST.

Description (Organism)	Sequence										%Identification	Accession
DY-8 peptide (*Z. cassumunar*)	1	**D**	**G**	**I**	**F**	**V**	**L**	**N**	**Y**	8		
cytosolic endo-b-N-acetylglucosaminidase 1-like (*Z. officinale*)	261	**D**	**G**	**I**	**F**	**V**	**-**	**N**	**Y**	267	88%	XP_042429169.1
hypothetical protein ZIOFF_004822 (*Z. officinale*)	275	**D**	**G**	**I**	**F**	**V**	**-**	**N**	**Y**	282	88%	KAG6531052.1
leucine-rich repeat receptor-like serine/threonine-protein kinase BAM1 (*Z. officinale*)	560	**S**	**G**	**I**	**S**	**V**	**L**	**N**	**Y**	567	75%	XP_042383899.1
villin-5-like (*Z. officinale*)	167	**D**	**G**	**I**	**F**	**I**	**L**	**D**	**T**	174	63%	XP_042407661.1
UNC93-like protein 1 (*Z. officinale*)	167	**L**	**I**	**P**	**F**	**V**	**L**	**N**	**Y**	174	63%	XP_042436030.1
