# Supplementary material for: A novel peptide derived from Zingiber cassumunar rhizomes exhibits anticancer activity against the colon adenocarcinoma cells (Caco-2) via the induction of intrinsic apoptosis signaling
Source: PLoS One. 2024 Jun 13;19(6):e0304701. doi: 10.1371/journal.pone.0304701 (PMC11175412; doi:10.1371/journal.pone.0304701)
Supplement: S4 Table — (PDF) [file pone.0304701.s006.pdf]

S4 Table Amino acid alignment of the IK-6 peptides in the homologous region as determined by Protein BLAST.

Description (Organism)	Sequence								%Identification	Accession
IK-6 peptide (*Z. cassumunar*)	1	I	P	T	D	E	K	6		
hypothetical protein ZIOFF_061232 (*Z. officinale*)	1081	V	P	T	D	E	K	1086	83%	KAG6477800.1
uncharacterized protein LOC122020010 isoform X1 (*Z. officinale*)	888	V	P	T	D	E	K	893	83%	XP_042433632.1
sulfoquinovosidase-like (*Z. officinale*)	502	V		T	D	E	K	507	83%	XP_042451933.1
twinkle homolog protein, chloroplastic/mitochondrial-like isoform X1 (*Z. officinale*)	373	I	P	T	E	E	K	378	83%	XP_042415854.1
probable disease resistance protein At1g61300 (*Z. officinale*)	62	I	P	T	D	E	V	67	83%	XP_042389958.1
