# Supplementary material for: A novel peptide derived from Zingiber cassumunar rhizomes exhibits anticancer activity against the colon adenocarcinoma cells (Caco-2) via the induction of intrinsic apoptosis signaling
Source: PLoS One. 2024 Jun 13;19(6):e0304701. doi: 10.1371/journal.pone.0304701 (PMC11175412; doi:10.1371/journal.pone.0304701)
Supplement: S1 Text — β-actin (rabbit polyclonal; 1: 500 dilution), anti-Bcl-2 (mouse monoclonal; 1: 500 dilution), anti-Bax (mouse monoclonal; 1: 1,000 dilution), anti-Caspase-3 (mouse monoclonal; 1: 500 dilution), anti-Cleavaged aspase-3 (mouse monoclonal; 1: 500 dilution), and anti-Caspase-9 (rabbit polyclonal; 1: 500 dilution). (PDF) [file pone.0304701.s007.pdf]

S1 Text List of used primary antibodies and the sources.

β-actin (rabbit polyclonal; 1: 500 dilution), anti-Bcl-2 (mouse monoclonal; 1: 500 dilution), anti-Bax (mouse monoclonal; 1: 1,000 dilution), anti-Caspase-3 (mouse monoclonal; 1: 500 dilution), anti-Cleavaged aspase-3 (mouse monoclonal; 1: 500 dilution), and anti-Caspase-9 (rabbit polyclonal; 1: 500 dilution)
