# Supplementary material for: A novel peptide derived from Zingiber cassumunar rhizomes exhibits anticancer activity against the colon adenocarcinoma cells (Caco-2) via the induction of intrinsic apoptosis signaling
Source: PLoS One. 2024 Jun 13;19(6):e0304701. doi: 10.1371/journal.pone.0304701 (PMC11175412; doi:10.1371/journal.pone.0304701)
Supplement: S1 Raw images — L: protein ladder, C: untreated cells, D: 0.5 μg/mL doxorubicin and DY-8 peptide concentration at 2.5 (P1), and 5 (P2) μM. (PDF) [file pone.0304701.s008.pdf]

S1 raw images. Original blot of protein expression of Bcl-2, Bax, caspase-9, cleaved caspase-3, caspase-3, and β-actin. L: protein ladder, C: untreated cells, D: 0.5 μg/mL doxorubicin and DY-8 peptide concentration at 2.5 (P_1), and 5 (P_2) μM.

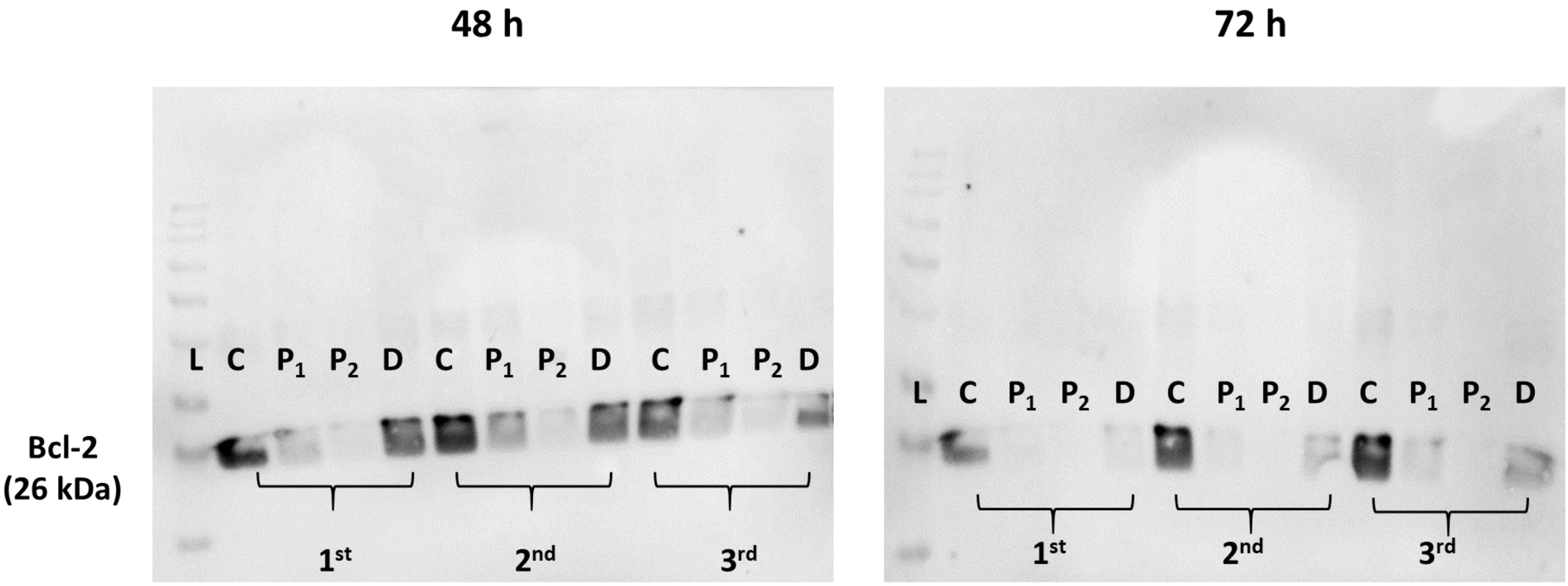

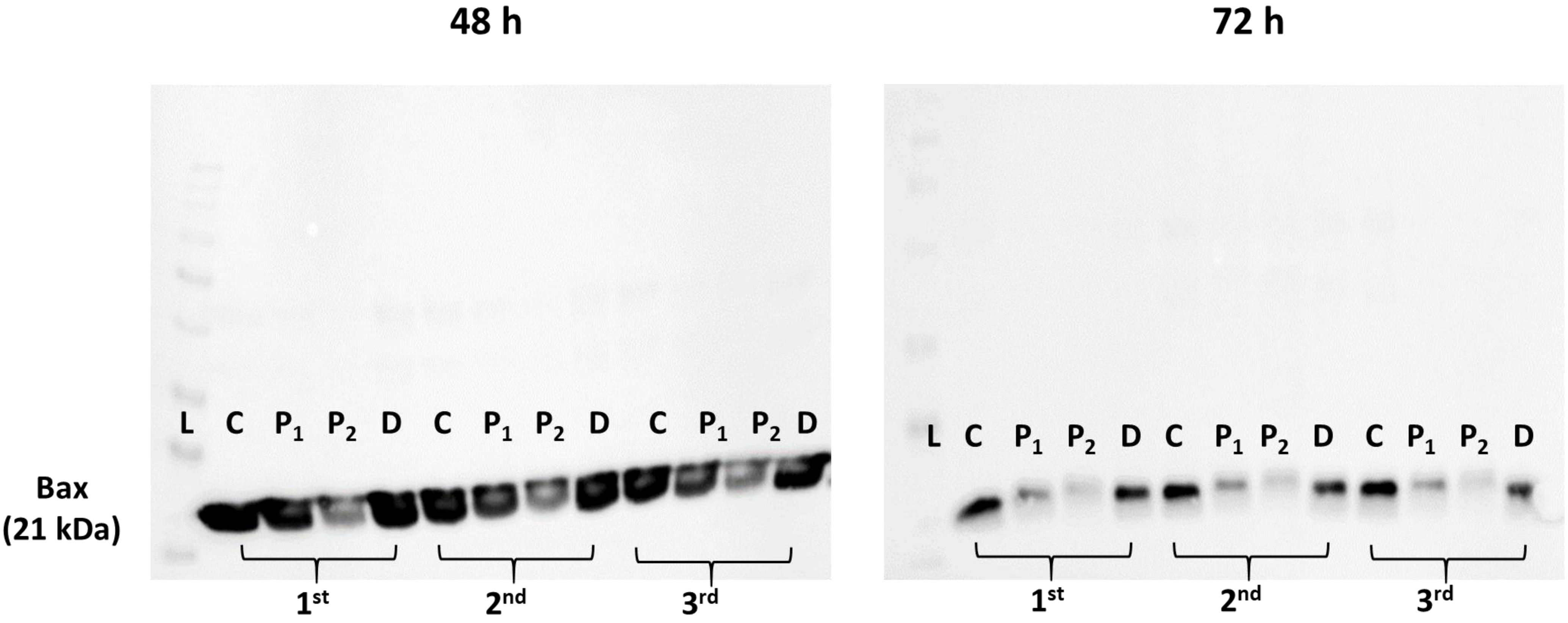
48 h
72 h
L C P1 P2 D C P1 P2 D C P1 P2 D
L C P1 P2 D C P1 P2 D C P1 P2 D
Bax
(21 kDa)
1st
2nd
3rd
1st
2nd
3rd

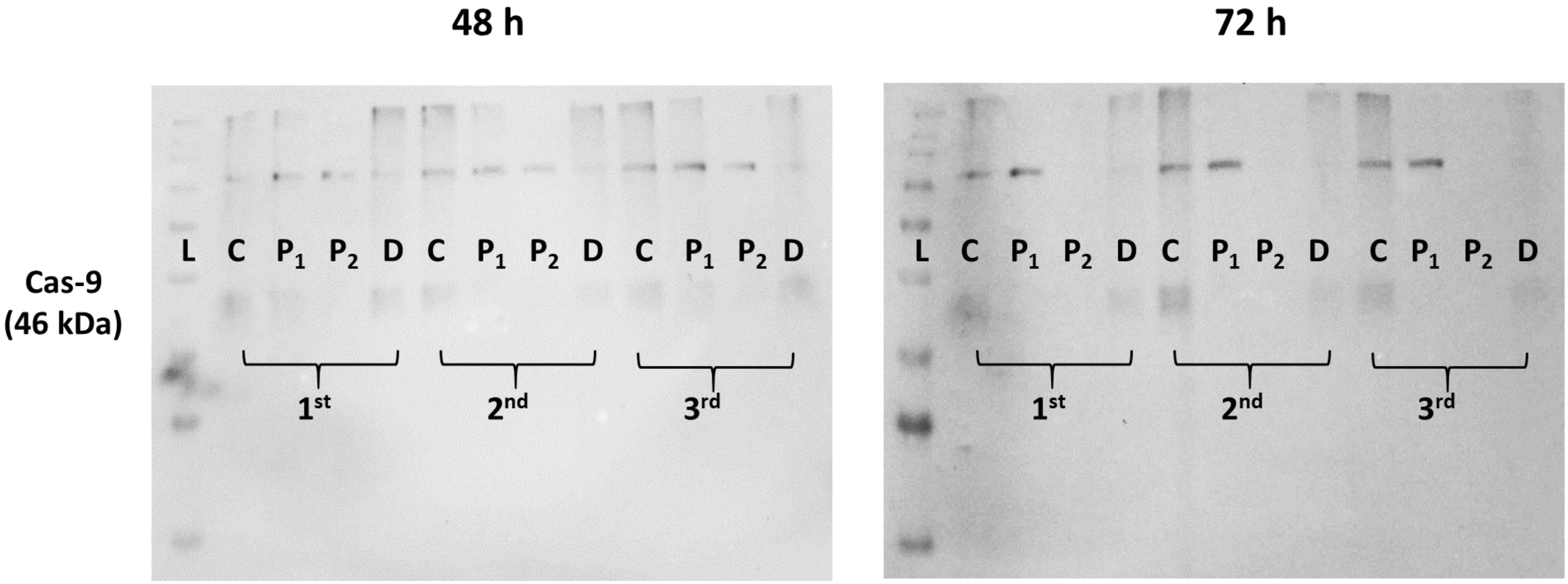
48 h
72 h
Cas-9
(46 kDa)
L C P_1 P_2 D C P_1 P_2 D C P_1 P_2 D
L C P_1 P_2 D C P_1 P_2 D C P_1 P_2 D
1st
2nd
3rd
1st
2nd
3rd

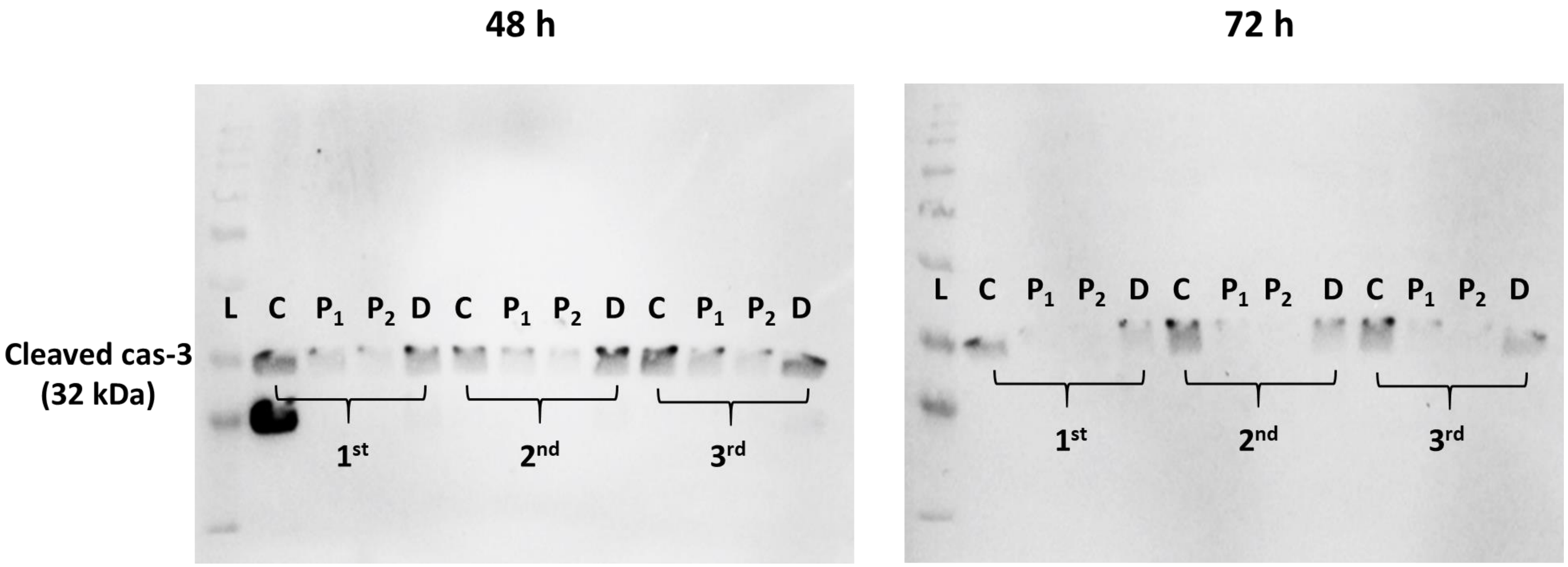
48 h
72 h
Cleaved cas-3
(32 kDa)
L C P1 P2 D C P1 P2 D C P1 P2 D
1st
2nd
3rd
L C P1 P2 D C P1 P2 D C P1 P2 D
1st
2nd
3rd

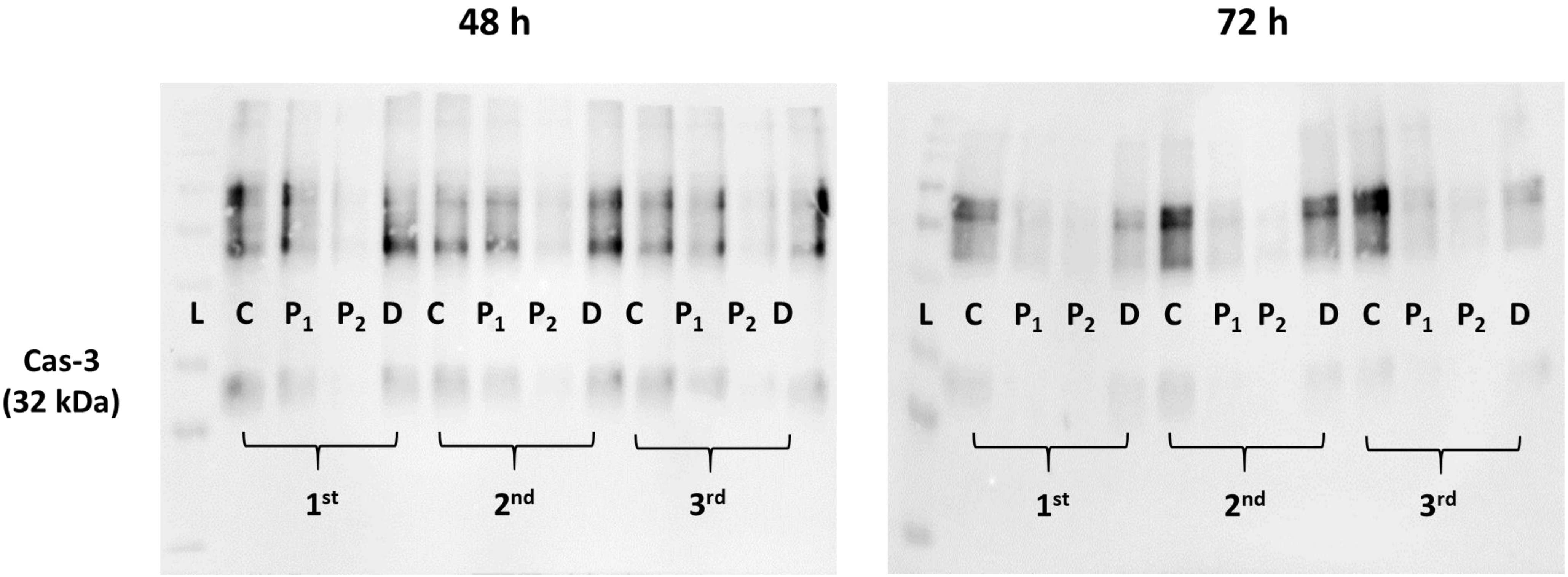
48 h
72 h
Cas-3
(32 kDa)
L C P1 P2 D C P1 P2 D C P1 P2 D
1st
2nd
3rd
L C P1 P2 D C P1 P2 D C P1 P2 D
1st
2nd
3rd

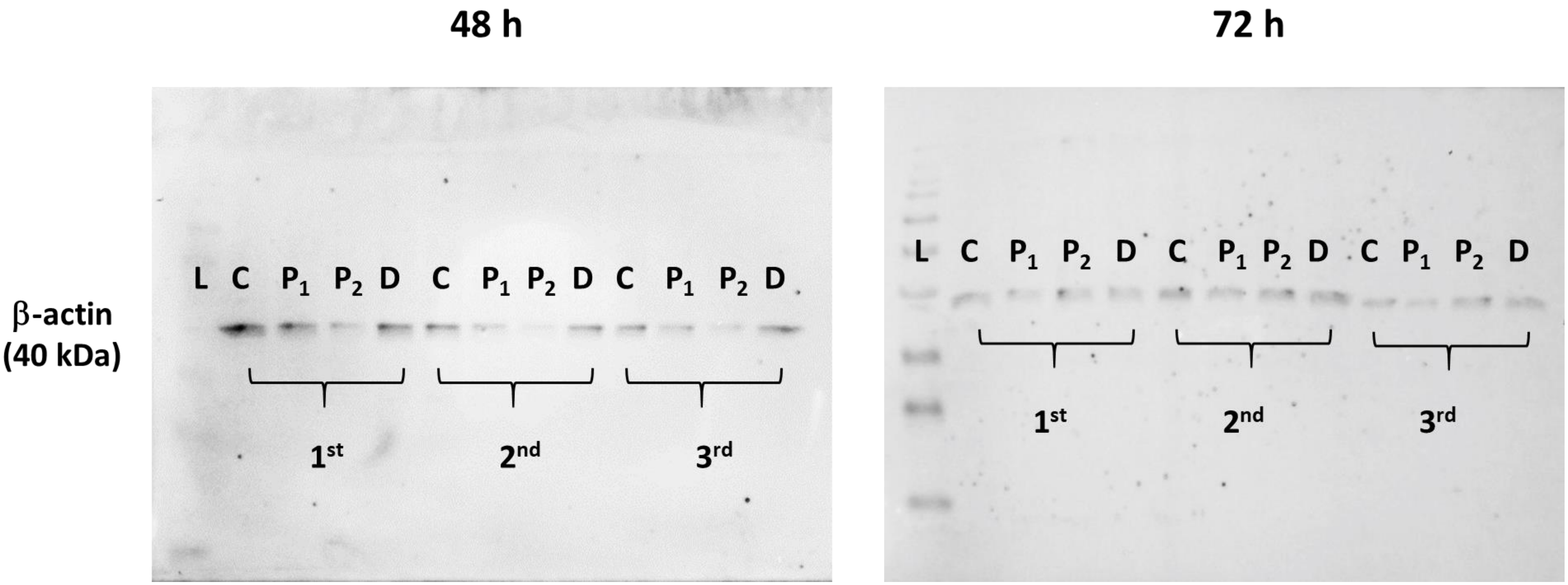
48 h
72 h
β-actin
(40 kDa)
L C P1 P2 D C P1 P2 D C P1 P2 D
1st
2nd
3rd
L C P1 P2 D C P1 P2 D C P1 P2 D
1st
2nd
3rd
